# Rejuvenation of Meiotic Cohesion in Oocytes during Prophase I Is Required for Chiasma Maintenance and Accurate Chromosome Segregation

**DOI:** 10.1371/journal.pgen.1004607

**Published:** 2014-09-11

**Authors:** Katherine A. Weng, Charlotte A. Jeffreys, Sharon E. Bickel

**Affiliations:** Department of Biological Sciences, Dartmouth College, Hanover, New Hampshire, United States of America; Cornell University, United States of America

## Abstract

Chromosome segregation errors in human oocytes are the leading cause of birth defects, and the risk of aneuploid pregnancy increases dramatically as women age. Accurate segregation demands that sister chromatid cohesion remain intact for decades in human oocytes, and gradual loss of the original cohesive linkages established in fetal oocytes is proposed to be a major cause of age-dependent segregation errors. Here we demonstrate that maintenance of meiotic cohesion in Drosophila oocytes during prophase I requires an active rejuvenation program, and provide mechanistic insight into the molecular events that underlie rejuvenation. Gal4/UAS inducible knockdown of the cohesion establishment factor Eco after meiotic S phase, but before oocyte maturation, causes premature loss of meiotic cohesion, resulting in destabilization of chiasmata and subsequent missegregation of recombinant homologs. Reduction of individual cohesin subunits or the cohesin loader Nipped B during prophase I leads to similar defects. These data indicate that loading of newly synthesized replacement cohesin rings by Nipped B and establishment of new cohesive linkages by the acetyltransferase Eco must occur during prophase I to maintain cohesion in oocytes. Moreover, we show that rejuvenation of meiotic cohesion does not depend on the programmed induction of meiotic double strand breaks that occurs during early prophase I, and is therefore mechanistically distinct from the DNA damage cohesion re-establishment pathway identified in G2 vegetative yeast cells. Our work provides the first evidence that new cohesive linkages are established in Drosophila oocytes after meiotic S phase, and that these are required for accurate chromosome segregation. If such a pathway also operates in human oocytes, meiotic cohesion defects may become pronounced in a woman's thirties, not because the original cohesive linkages finally give out, but because the rejuvenation program can no longer supply new cohesive linkages at the same rate at which they are lost.

## Author Summary

Meiosis is a specialized type of cell division that gives rise to sperm and eggs. In a woman's thirties, errors in meiotic chromosome segregation rise exponentially, significantly increasing the probability that she will conceive a fetus with Down Syndrome (Trisomy 21). Accurate chromosome segregation during meiosis depends on protein linkages (cohesion) that hold sister chromatids together. The widely held view is that under normal conditions, cohesion can only be established during DNA replication, and the original cohesive linkages formed in fetal oocytes are gradually lost as a woman ages. However, it seems unlikely that the same cohesion proteins could survive for even five years, much less 25 years. Here we show that Drosophila oocytes possess an active rejuvenation program that is required to load newly synthesized cohesion proteins and to establish new cohesive linkages after meiotic DNA replication. When we reduce the proteins responsible for rejuvenation after meiotic S phase, cohesion is lost and meiotic chromosomes missegregate. If such a rejuvenation pathway also exists in human oocytes and becomes less efficient with age, oocytes of older women may no longer be able to replace cohesive linkages at the same rate that they are lost.

## Introduction

In both mitotic and meiotic cells, sister chromatid cohesion is required for accurate chromosome segregation, and the cohesive linkages that hold sister chromatids together depend on the cohesin complex which forms a DNA-entrapping ring [1], [2]. In addition to holding sister chromatids together, cohesion plays several additional essential roles during meiosis. The integrity of the synaptonemal complex, a meiosis-specific structure that holds homologs in close proximity during recombination, depends on cohesion proteins and crossovers between homologs are reduced in cells in which cohesion is compromised [3]. In addition, cohesion along the arms of sister chromatids provides an evolutionarily conserved mechanism that keeps recombinant homologs physically associated until anaphase I [4]–[6]. By maintaining chiasmata, arm cohesion promotes proper orientation and microtubule attachments of homologous chromosomes on the metaphase I spindle and is therefore crucial for accurate segregation of homologs during the first meiotic division.

The timeline of human oogenesis presents a daunting challenge for the maintenance of meiotic cohesion [7]. Oocytes undergo meiotic DNA replication, establish sister chromatid cohesive linkages and complete meiotic recombination during fetal development. Before birth, oocytes enter a prolonged diplotene arrest (known as dictyate), and resumption of meiosis occurs only as individual oocytes are recruited for ovulation. Because the majority of human oocytes remain arrested for decades, the continued physical association of recombinant homologs and their accurate segregation during meiosis I demands that cohesion along the arms of sister chromatids remain intact during this extended timeframe.

Chromosome segregation errors during female meiosis are the leading cause of miscarriages and birth defects in humans [8]. Furthermore, the risk of producing aneuploid gametes increases exponentially as women age. A correlation between advanced maternal age and increased incidence of single chromatids prior to the second meiotic division has been reported for human oocytes obtained from cancer patients and from women undergoing in vitro fertilization [9], [10]. While the mechanisms underlying the maternal age effect are likely to be complex, work in Drosophila and mice also indicates that meiotic cohesion weakens with age and supports the hypothesis that deterioration of meiotic cohesion plays an important role in age-related segregation errors in human oocytes [6], [11]–[14].

Based on work in budding yeast, it is widely accepted that under normal conditions, cohesive linkages are only established during S phase [1], [2]. However, if this were the case for human oocytes, the same cohesin complexes used for cohesion establishment in the human fetal ovary still would be present in adult oocytes years later. On first reflection, this provides a satisfying explanation for why cohesion defects would be more prevalent in the oocytes of older women. However, is it really possible that the same cohesin rings remain intact on meiotic chromosomes for even five years, much less 25? An alternate possibility is that maintenance of meiotic cohesion is an active process that utilizes a specialized rejuvenation program to establish new cohesive linkages throughout the extended timeframe of prophase I. Precedence for cohesion establishment outside of S phase comes from work in budding yeast, which has demonstrated that under certain conditions vegetative cells can establish functional cohesive linkages during G2 [15]–[19]. Moreover, in both Drosophila and mouse oocytes, localization of the cohesin loader Nipped-B along the arms of meiotic chromosome during pachytene has been observed [20]–[22], consistent with the possibility that cohesin complexes are loaded and converted to functional linkages during meiotic prophase.

Here we utilize Drosophila to test the hypothesis that cohesion rejuvenation occurs during meiotic prophase. The Drosophila oocyte provides an excellent system to study the maintenance of meiotic cohesion because prophase I lasts approximately six days [23], and the linear array of oocytes within each of the ovarioles comprising the ovary permits one to monitor chromosome morphology at progressive stages during meiotic prophase. In addition, a simple genetic assay allows us to measure the fidelity of meiotic chromosome segregation. We have used a Gal4/UAS inducible RNAi strategy to ask whether cohesion defects occur if we reduce cohesion regulators or cohesin complex subunits after meiotic cohesion is established normally during meiotic S phase. We find that a rejuvenation program operating during prophase I is necessary to sustain a level of meiotic cohesion that is sufficient for chiasma maintenance and accurate chromosome segregation. Our data support the model that rejuvenation of meiotic cohesion requires Nipped-B-dependent loading of newly synthesized cohesin complexes and Eco-dependent establishment of new cohesive linkages. Furthermore, Eco-mediated cohesion rejuvenation does not depend on induction of double-strand breaks and therefore differs from the damage-induced cohesion re-establishment pathway that operates in yeast cells. We raise the possibility that the rejuvenation pathway we have uncovered in Drosophila oocytes may represent an evolutionarily conserved mechanism to ensure that an adequate number of cohesive linkages remain present during the extended period that metazoan oocytes stay arrested in prophase I.

## Results

### The matα driver induces Eco knockdown after meiotic S phase

We reasoned that if it were to occur, rejuvenation of cohesion during the extended prophase I period might utilize factors that are normally required for cohesion establishment. Therefore, we began our analysis by focusing on the cohesion establishment factor Eco, (also known as Deco, Drosophila Eco1 [24]). In yeast, Eco1 acetyltransferase activity is required to establish cohesion during S phase [1], [2].

In order to ask whether Eco activity is required to keep meiotic cohesion intact after its original establishment, we employed a Gal4/UAS inducible RNAi strategy that allowed us to leave Eco levels and activity unaffected during meiotic S phase, when cohesion is established, and to induce RNAi-mediated decay of Eco transcripts only after cohesive linkages are generated. To accomplish this, we used the mat-α-tubulin-Gal4-VP16 driver (hereafter abbreviated matα driver) which has previously been shown to begin expression during mid-prophase [25]. Using a UASp-Actin-GFP reporter, we verified that the matα driver is not active until after meiotic DNA replication and would therefore allow us to induce Eco knockdown after normal cohesion establishment (Figure S1).

We used the matα driver to induce expression of a UAS-Eco RNAi hairpin transgene (VDRC.Eco.35982, Table S1, hereafter referred to as Eco RNAi^GD^) and performed single molecule FISH [26] to quantify the number of Eco transcripts in control and Eco knockdown (KD) germline cysts at different stages during oogenesis (Figure S2). In germarial region 3, the number of Eco transcripts per area for control and Eco KD was the same (p = 0.95, Figure S2B), confirming that Eco knockdown does not commence until after meiotic S phase. We are confident that our assay is sensitive enough to detect a change in the number of Eco transcripts in the germarium because we observed a significant reduction (∼14%) in region 3 for the viable allelic combination *eco^1^/eco^2^* compared to the control (p = 0.018, Fig S2B). At later stages, when matα driver expression is more robust, Eco transcripts were significantly reduced in Eco RNAi^GD^ egg chambers, confirming that matα driving Eco RNAi^GD^ reduces Eco transcripts in germline cells, but only after cohesive linkages are established during meiotic S phase.

### Eco function is required after meiotic S phase

**Figure 1:**
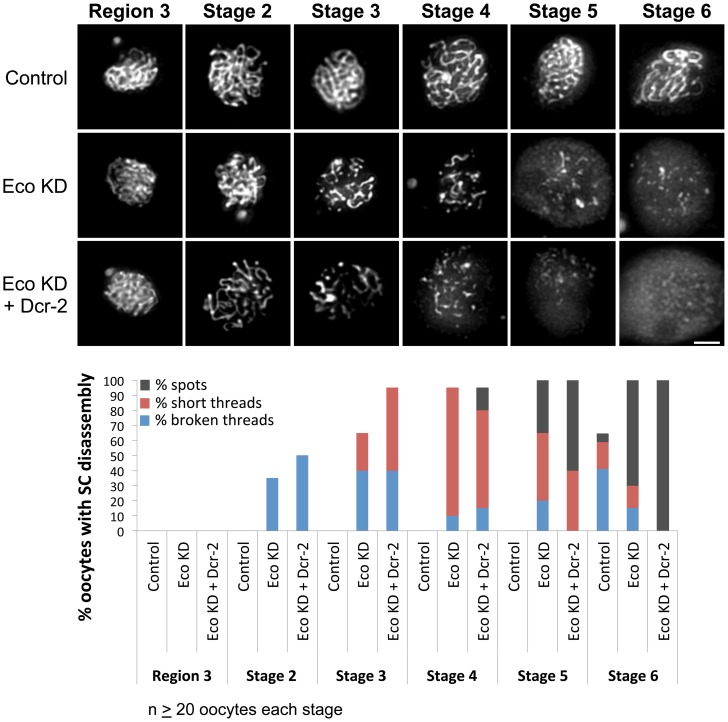
Eco knockdown in mid-prophase causes premature disassembly of the SC. (Top) C(3)G immunostaining is shown for oocytes from females containing the UAS-Eco RNAi^GD^ transgene in the absence of driver (Control), the presence of the matα driver (Eco KD) and the presence of the matα driver and a UAS-Dicer-2 transgene to increase Eco RNAi efficiency (Eco KD + Dcr-2). Long continuous threads of SC are apparent in Control oocytes until stage 6. In contrast, premature disassembly of the SC is visible in Eco KD and Eco KD + Dcr-2 oocytes starting as early as stage 2. Images for each stage were captured and processed identically and projections of deconvolved Z-series are shown. Scale bar, 2 µm. (Bottom) Quantification of SC defects is presented. Oocytes with SC disassembly were assigned to one of three categories with increasing severity: broken threads, short threads and spots. In Control oocytes, SC disassembly is not detected until stage 6. In contrast, premature disassembly of the SC is detectable beginning at stage 2 in the Eco KD oocytes. As prophase I progresses, both the severity of the defects and the percentage of oocytes affected increase. When Dicer-2 is overexpressed, the phenotype is enhanced at all stages, in both the percentage of oocytes with defects and the severity of defects. At least 20 oocytes were scored for each genotype at each stage.

To begin to explore whether Eco activity is required to maintain meiotic cohesion, we investigated whether reduction of Eco during meiotic prophase impacts the integrity of the synaptonemal complex (SC), a tripartite proteinacious structure that holds homologous chromosomes in close proximity during the process of meiotic recombination [27]. Mutations in cohesion proteins have been shown to disrupt the formation and/or maintenance of the SC [28]–[35]. In wild-type Drosophila oocytes, full-length SC forms in region 2A of the germarium and remains intact until stage 6 [36], [37]. To monitor SC stability in Eco KD oocytes, we stained for the SC transverse filament protein C(3)G [36], [38]. In control ovarioles (Eco RNAi^GD^ transgene, no driver), we observed long continuous C(3)G threads until stage 6 (Figure 1) when normal SC disassembly occurs at the end of pachytene. However, when Eco is knocked down in mid-prophase I (matα driving Eco RNAi^GD^ transgene), we observed a number of SC defects. Figure 1 shows representative images of the SC in control and Eco KD oocytes at different stages, as well as quantification of the defects we observed. In order to quantify the severity of the defects, we utilized three categories (broken threads, short threads, or spots) to describe oocytes that lacked normal, continuous C(3)G threads. These categories represent the range of defects we observed, with spots corresponding to the most severe disruption of the SC.

When Eco is knocked down during mid-prophase I, the SC appears normal in all germarial stages (regions 2A, 2B and 3), but premature disassembly is visible beginning at stage 2 (Figure 1, Eco KD), after expression of the matα driver begins. The majority of stage 2 oocytes contain continuous SC, but minor defects (broken threads) are apparent in approximately 35% of the Eco KD oocytes. By stage 3, the majority of oocytes exhibit SC defects; the percentage of oocytes with broken threads increases to 40%, while 25% display a more severe phenotype (short threads). As oocytes progress through prophase I, the severity of the defects increases. By stage 4, premature disassembly of the SC is visible in all Eco KD oocytes examined, the majority of which contain only short threads of SC. At stage 5, 35% of the Eco KD oocytes contain only spots of SC signal, and the majority of the stage 6 oocytes belong to this category (Figure 1). These results demonstrate that when Eco is knocked down after S phase, progressive deterioration of the SC occurs.

Because the Drosophila germline is somewhat refractory to RNAi [39] and because the Eco RNAi^GD^ vector (modified pUAST vector pMF3) is not efficiently expressed in the germline [40], [41], we overexpressed Dicer-2 [41] to increase the efficacy of the Eco RNAi^GD^ hairpin. Dicer-2 is a component of siRNA-dependent RISC (RNA induced silencing complex) and is required for siRNA-mediated silencing [42]. As shown in Figure 1, SC defects are enhanced both in the number of oocytes affected and the severity of the defects when the matα driver induces overexpression of a UAS-Dicer-2 transgene simultaneously with Eco RNAi^GD^. Overall, these data argue that Eco activity during meiotic prophase is essential to stabilize the synaptonemal complex and are consistent with a role for Eco in maintaining meiotic cohesion after meiotic S phase.

### Meiotic chromosomes missegregate when Eco is reduced after S phase

**Figure 2:**
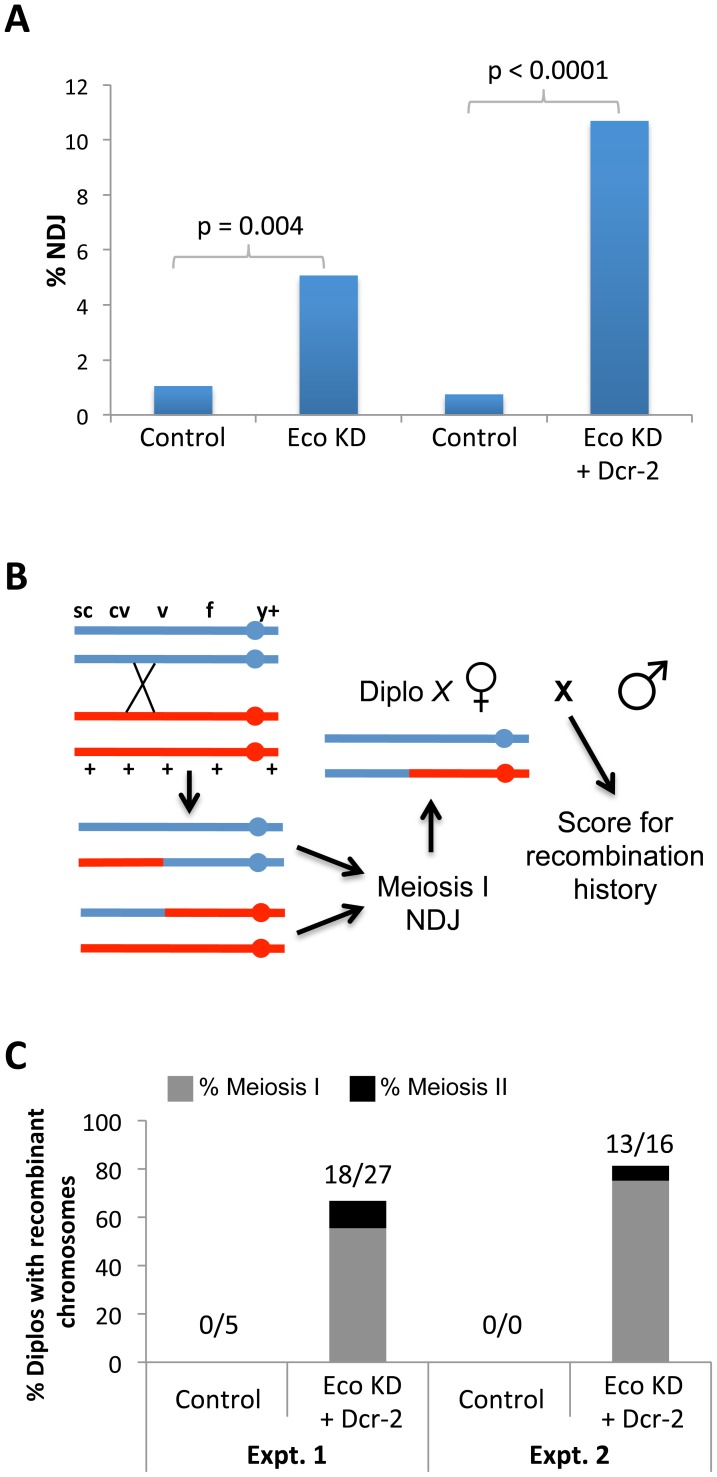
Chiasmata are not maintained if Eco is knocked down during mid-prophase I. (A) *X* chromosome nondisjunction (NDJ) is significantly higher (p = 0.004) in oocytes expressing the UAS-Eco RNAi^GD^ transgene under the control of the matα driver (Eco KD, n = 631) compared to oocytes containing the UAS-Eco RNAi^GD^ transgene in the absence of driver (Control, n = 763). Overexpression of the UAS-Dicer-2 transgene in Eco KD oocytes (Eco KD + Dcr-2, n = 505) causes a further increase in *X* chromosome NDJ (p<0.0001) compared to UAS-Eco RNAi^GD^ + UAS-Dicer-2 oocytes lacking the matα driver (Control, n = 799). (B) The method used to determine the recombinational history of *X* chromosomes that missegregate is shown. The NDJ test utilizes females that are heterozygous for several visible markers on the *X* chromosome, including a centromere-linked y^+^ marker. In this illustration, a single crossover between *cv* and *v* produces a bivalent with two recombinant and two non-recombinant chromatids. Subsequent meiosis I NDJ could result in a Diplo-*X* female that inherits two *X* chromosomes from her mother, only one of which has undergone a crossover. In order to determine the genotype of the *X* chromosomes in the Diplo-*X* female, she is crossed to a wild-type male and her sons are scored for the *X* chromosome visible markers. Because the two largest classes of her progeny will correspond to parentals, the genotype of the *X* chromosomes in the Diplo-X female can be deduced, allowing us to determine whether recombinant chromosomes missegregated in the NDJ test. (C) The results of two independent experiments are presented. Each utilized females containing the UAS-Eco RNAi^GD^ and UAS-Dicer-2 transgenes in the absence of driver (Control) and the presence of the matα driver (Eco KD + Dcr-2). In both tests, more than 66% of Diplo-*X* progeny result from missegregation of recombinant chromosomes (18 out of 27, and 13 out of 16 Diplo-*X* females, respectively). Moreover, 83% of the recombinant bivalents that missegregate result in Diplo-*X* females that are heterozygous for the y^+^ centromere-linked marker and therefore represent meiosis 1 NDJ events (15 out of 18, and 12 out of 13 Diplo-*X* females, respectively). See Figure S7 for raw data.

If Eco were required to rejuvenate meiotic cohesion after S phase, we would expect RNAi-mediated reduction of Eco activity during prophase to result in chromosome segregation errors. Therefore, we used our standard genetic assay (see Materials and Methods) to measure the fidelity of chromosome segregation in Eco KD and control oocytes. Induction of the Eco RNAi^GD^ transgene with the matα driver caused a significant increase in meiotic nondisjunction (p = 0.004, Figure 2A), and chromosome segregation errors increased even more substantially in Eco KD oocytes when Dicer-2 was over-expressed (p<0.0001, Figure 2A). Our findings that chromosome segregation errors in Drosophila oocytes increase when Eco is reduced during prophase I indicate that Eco activity is required after S phase to ensure accurate chromosome segregation during meiosis.

We carried out a number of controls to rule out nonspecific effects and confirm that the SC and segregation defects we observed in Eco KD oocytes were indeed due to reduction of Eco during meiotic prophase. Expression of two additional Eco hairpin transgenes (Eco RNAi^V1^ and Eco RNAi^V22^, see Table S1) with the matα driver caused premature disassembly of the SC (Figure S3), and induction of the Eco RNAi^V1^ hairpin also resulted in meiotic NDJ (Figure S3). The low fertility of Eco RNAi^V22^ KD females (even in the absence of UAS-Dcr-2) prevented us from measuring meiotic NDJ in this genotype. However, together these data verify that the effects of Eco RNAi^GD^ transgene expression were not due to nonspecific targets. In addition, in oocytes in which matα-induced Dicer-2 overexpression occurred in the absence of any RNAi hairpin transgene, we observed normal SC (Figure S4A) and no significant increase in NDJ (Figure S4B) confirming that the enhanced SC and NDJ defects we observed when Dicer-2 was overexpressed in a Eco KD background were a result of the increased RNAi efficiency. Finally, we also validated that the onset of SC defects at stage 2 in Eco KD oocytes (Figure 1) occurred as a natural consequence of the temporal expression of the matα driver and not because the germarium is refractory to RNAi. When we used the nanos-Gal4-VP16 driver [43] to induce the expression of Eco RNAi^GD^ in the germarium, we observed premature disassembly of the SC beginning in germarial region 2B (Figure S5A & B), providing evidence that Eco knockdown in the germarium can be achieved with the nanos driver. Moreover, knockdown of Eco using the nanos driver resulted in a significant increase in segregation errors (p<0.0001, Figure S5C). Most likely, nanos-driven knockdown of Eco impacts the establishment of cohesion during meiotic S phase as well as any prophase I functions of Eco protein.

### Chiasmata are not maintained when Eco is knocked down during mid-prophase

One prerequisite for accurate segregation during meiosis I is that homologous chromosomes must remain physically associated until anaphase I when they segregate to opposite poles. After crossovers are formed, it is cohesion along the arms of sister chromatids that keeps recombinant homologs tethered to each other and chiasmata stabilized [4]–[6]. If knockdown of Eco after meiotic S phase causes loss of cohesion during prophase, we would expect chiasmata to become destabilized and recombinant chromosomes to missegregate during anaphase I. Therefore, we utilized a genetic assay [11] that allowed us to monitor for loss of chiasma maintenance in Eco KD oocytes.

We first verified that crossover formation was not severely disrupted when Eco RNAi^GD^ was induced with the matα driver in combination with Dicer-2 overexpression. We monitored crossover frequency in four intervals along the *X* chromosome in Eco KD and control oocytes and found no significant difference between the two genotypes (Figure S6), indicating that crossovers form normally when Eco is reduced during mid/late pachytene.

To obtain direct evidence that Eco KD during prophase I causes loss of cohesion, we assayed the recombinational history of the missegregating chromosomes in Eco RNAi^GD^ oocytes. To obtain these data, we performed a standard NDJ test using Eco KD and control females heterozygous for an *X* chromosome with several visible markers, including one located proximal to the centromere (Figure 2B). By performing an additional cross with the Diplo-*X* progeny arising from the NDJ test, we were able to determine what fraction of missegregating chromosomes had undergone one or more crossovers and, based on the centromere-linked y^+^ marker, whether segregation errors occurred primarily in meiosis I or in meiosis II.

The results from two independent experiments shown in Figure 2C demonstrate that the majority of bivalents that exhibit segregation defects in Eco KD oocytes are recombinant and that segregation errors occur primarily during meiosis I. In the first experiment, 18 of the 27 Diplo-*X* females arising from chromosome NDJ in Eco KD oocytes harbored at least one recombinant chromosome. In the second experiment, 13 of the 16 Diplo-*X* females contained at least one recombinant chromosome. In addition, because 27 of the 31 Diplo-*X* females harboring recombinant chromosomes were heterozygous for the centromere proximal y^+^ marker (Experiments 1 and 2 combined, see Figure S7), we conclude that the majority of segregation errors arose from meiosis I NDJ (Figures 2C and S7). Missegregation of recombinant chromosomes during meiosis I supports the hypothesis that when Eco is knocked down after meiotic S phase, crossovers are formed but chiasmata are not stabilized due to loss of arm cohesion. Moreover, it is important to note that our assay underrepresents the percentage of recombinant bivalents that missegregate, because it is possible for a Diplo-*X* female to inherit two non-recombinant chromatids from a recombinant bivalent (Figure 2B). Together, these results demonstrate that Eco activity is required to maintain meiotic cohesion after cohesive linkages are formed during S phase and that Eco-mediated rejuvenation of cohesion during meiotic prophase is necessary for chiasma maintenance and accurate chromosome segregation.

### Synthesis of cohesin subunits is required during meiotic prophase I to maintain cohesion

Our analysis of Eco KD oocytes indicates that Eco activity is required to maintain cohesive linkages during meiotic prophase I. One possibility is that re-acetylation of SMC3 molecules within existing cohesive rings is required to stabilize meiotic cohesion during the prolonged period of prophase I. Alternatively, Eco-mediated rejuvenation of cohesion during prophase I could involve establishment of new linkages. Matα-driven knockdown of cohesin subunits should only impact meiotic cohesion if the latter were true. Therefore, we used the matα driver to induce expression of SMC1 RNAi^V22^, SMC3 RNAi^V20^, or Stromalin (SA) RNAi^V20^ hairpins (see Table S1) to reduce synthesis of cohesin subunits after establishment of meiotic cohesion. These transgenic constructs utilized the Valium 20 or Valium 22 vectors optimized for expression in the Drosophila germline [39].

**Figure 3:**
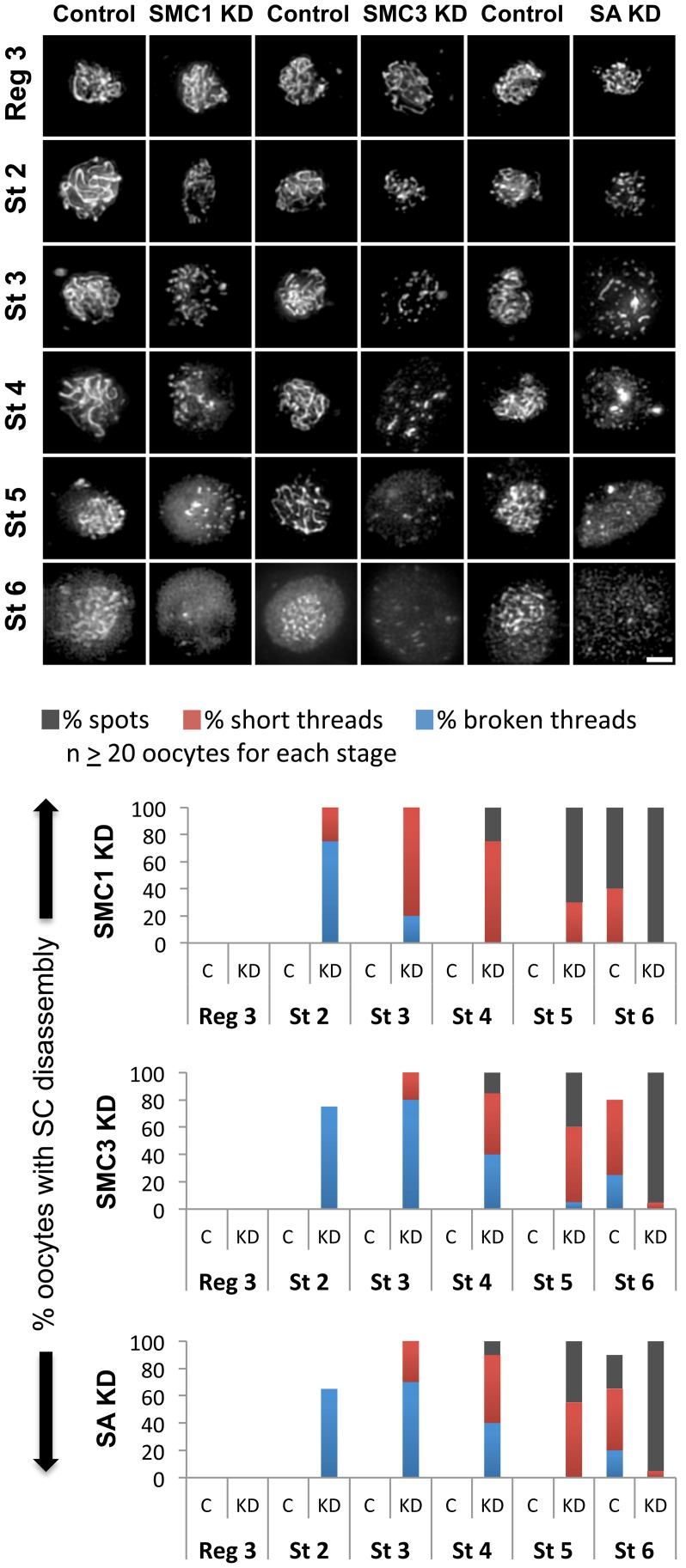
Knockdown of cohesin subunits in mid-prophase causes SC defects. (Top) C(3)G immunostaining is shown for oocytes in which expression of the UAS-SMC1 RNAi^V22^, UAS-SMC3 RNAi^V20^ or UAS-SA RNAi^V20^ transgene is induced by the matα driver (SMC1 KD, SMC3 KD, SA KD) as well as a control for each in which the respective RNAi transgene is present but the driver is absent. In contrast to the long continuous threads visible in control oocytes until stage 6, premature disassembly of the SC is visible in SMC1 KD, SMC3 KD, and SA KD oocytes starting as early as stage 2. Images for each stage were captured and processed identically. Projections of deconvolved Z-series are shown. Scale bar, 2 µm. (Bottom) Quantification of SC defects from region 3 (Reg 3) through stage 6 (St 6) is presented for the control (C) and knockdown (KD) genotypes described above. SC disassembly is not detected until stage 6 in Control oocytes for each RNAi transgene. In contrast, premature disassembly of the SC is detectable beginning at stage 2 (St 2) in the SMC1 KD, SMC3 KD, and SA KD oocytes. As prophase I progresses, both the severity of the defects and the percentage of oocytes affected increase. At least 20 oocytes were scored for each genotype at each stage.

Like reduction of Eco during meiotic prophase, knockdown of SMC1, SMC3 or SA using the matα driver results in premature disassembly of the SC starting at stage 2 (Figure 3). A comparison of each KD genotype with its corresponding control genotype (UAS RNAi, no driver) is shown in Figure 3. Similar to what we observed for Eco KD oocytes, both the number of oocytes affected and the severity of the defects escalate as cohesin knockdown oocytes progress through prophase I. In addition, the phenotype at each stage of oogenesis is very similar for each of the cohesin knockdowns. These data indicate that synthesis of new cohesin subunits is required during meiotic prophase I to keep the SC intact.

**Figure 4:**
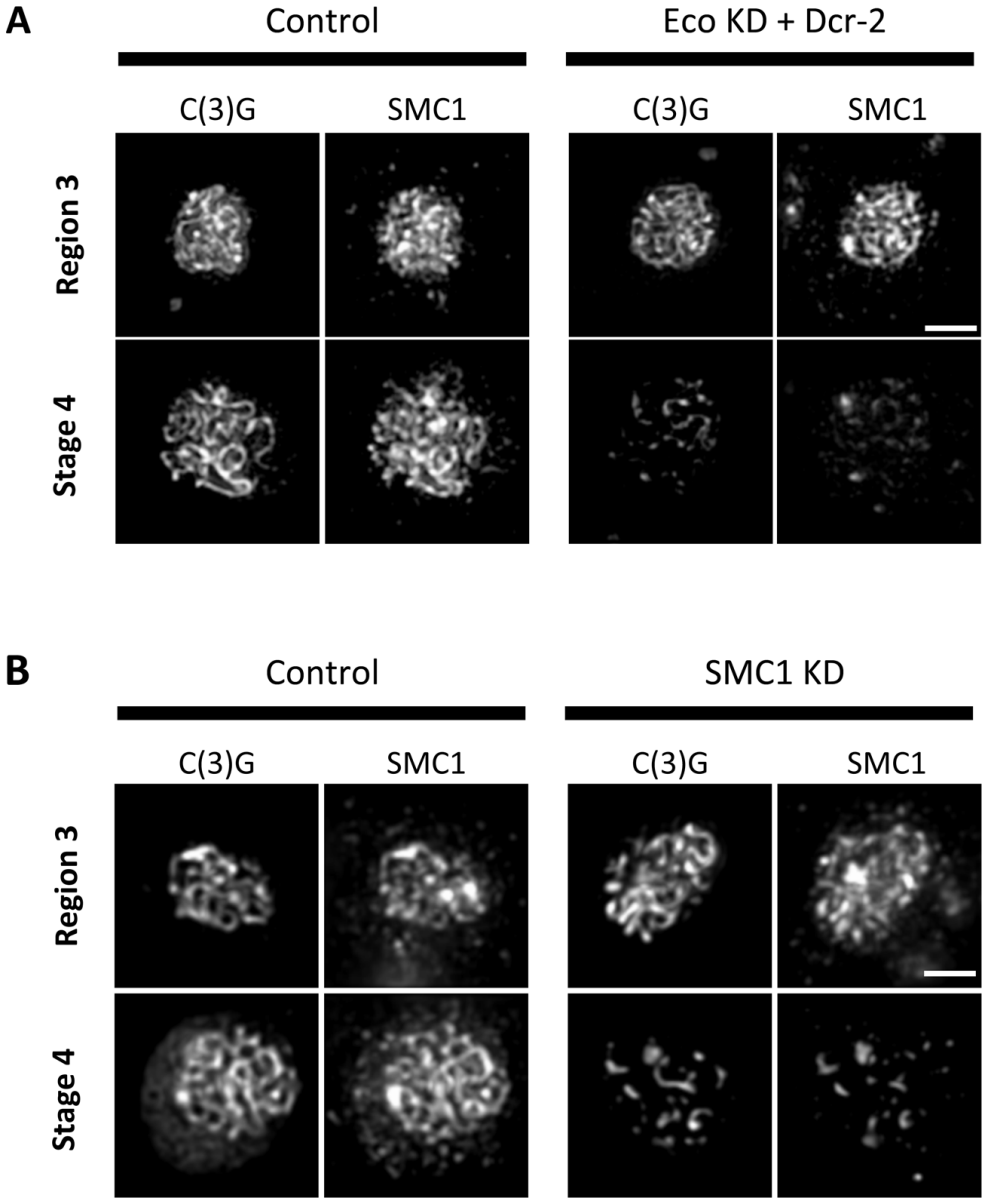
Maintenance of chromosome cores during pachytene requires Eco activity and synthesis of SMC1. (A) SMC1 and C(3)G immunostaining is shown for Region 3 and Stage 4 oocytes from females containing the UAS-Eco RNAi^GD^ and UAS-Dicer-2 transgenes in the absence (Control) and presence of the matα driver (Eco KD + Dcr-2). In Region 3, for both Control and Eco KD + Dcr-2 oocytes, long continuous C(3)G and SMC1 threads are apparent, indicating that SMC1 chromosome cores are intact. However, by stage 4, severe fragmentation of C(3)G and SMC1 threads is evident in Eco KD oocytes. (B) SMC1 and C(3)G immunostaining is shown for Region 3 and Stage 4 oocytes from females containing the UAS-SMC1 RNAi^V22^ transgene in the absence (Control) and presence of the matα driver (SMC1 KD). Long continuous threads of C(3)G and SMC1 are visible in Region 3 for both Control and SMC1 KD oocytes. In contrast, the signal for both proteins is limited to short stretches and spots in SMC1 KD oocytes at Stage 4, consistent with loss of cohesin from chromosome cores. Images in both A and B are projections of deconvolved Z-series and for each antibody. Control and KD images for each stage were captured and processed identically. Scale bar, 2 µm.

Given the similarity of SC defects in Eco KD and cohesin KD oocytes, we asked whether chromatin localization of SMC1 was perturbed when Eco or SMC1 proteins were reduced after meiotic S phase. One possibility is that premature disassembly of the SC occurs in these genotypes because of disruption of the cohesin-enriched chromosome cores that form a scaffold for the axial elements of the SC [29], [35], [44], [45]. We performed C(3)G and SMC1 co-immunolocalization experiments with ovarioles of Eco KD and SMC1 KD females and their respective controls (no driver) and compared region 3 oocytes (before SC defects occur) with those at stage 4 (when SC defects are pronounced). In both Eco KD and SMC1 KD oocytes, long continuous SMC1 threads were visible in region 3 oocytes, coincident with intact SC (Figure 4). However, in both genotypes, the SMC1 signal was restricted to short threads and spots in stage 4 oocytes, similar to that of the C(3)G signal. This pattern contrasts sharply with the extensive SMC1 threads visible in stage 4 oocytes for both control genotypes (Figure 4). Our findings indicate that SMC1 protein synthesis and Eco activity are required after meiotic S phase to maintain cohesin-enriched chromosome cores during pachytene. These data support the model that chromatin association of newly synthesized cohesin subunits occurs during prophase I and depends on Eco.

**Figure 5:**
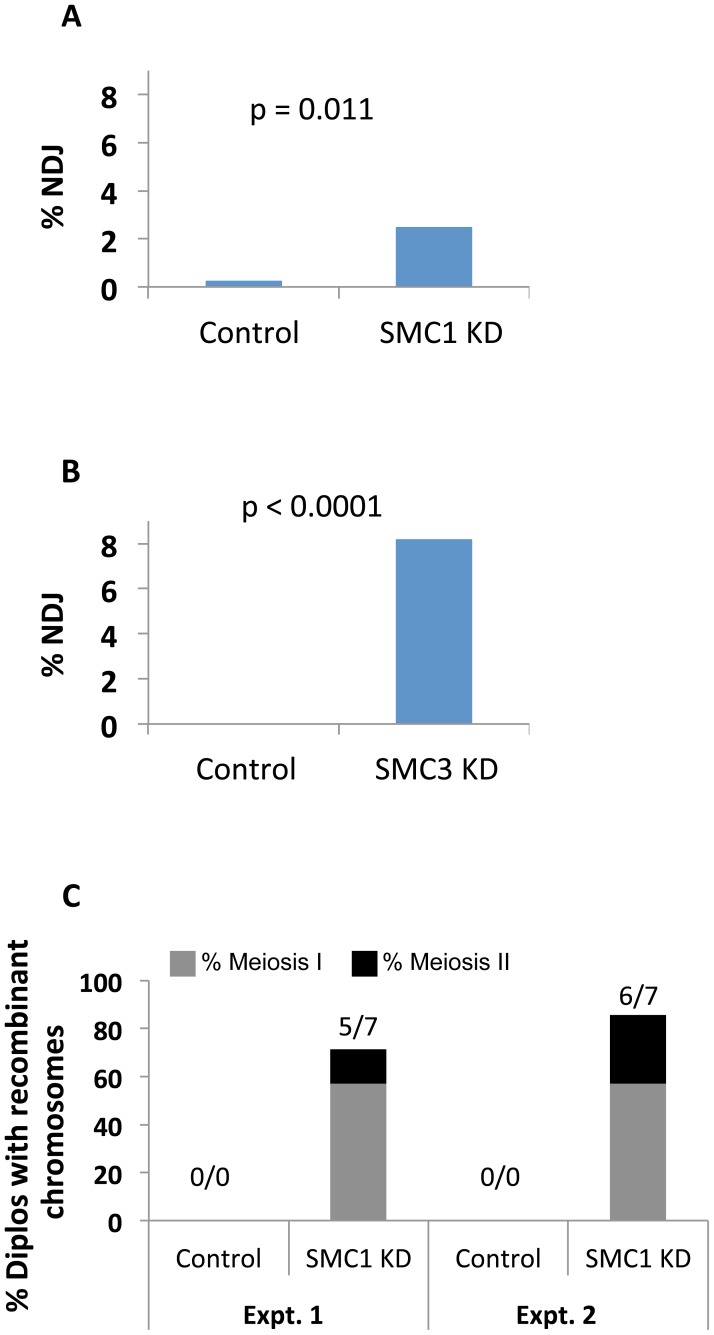
Cohesin subunit knockdown after meiotic S phase causes loss of chiasmata and chromosome segregation defects. (A) The UAS-SMC1 RNAi^V22^ transgene driven by matα-Gal4-VP16 (SMC1 KD) resulted in 2.48% *X* chromosome NDJ (n = 725), significantly higher than that observed in UAS-SMC1 RNAi^V22^ oocytes lacking the matα driver (Control, 0.27%, n = 730). (B) Expression of the UAS-SMC3 RNAi^V20^ transgene with the matα driver (SMC3 KD) caused 8.21% NDJ (n = 682), significantly higher than that observed in females containing the UAS-SMC3 RNAi^V20^ transgene in the absence of driver (Control, 0% NDJ, n = 810). (C) In two independent tests, at least 71% of Diplo-*X* females arising from SMC1 RNAi^V22^ KD oocytes contained recombinant chromosomes (5 out of 7, and 6 out of 7 respectively). Of these 11 Diplo-*X* females, the majority (4 out of 5, and 4 out of 6 respectively) contained *X* chromosomes that were heterozygous for the y^+^ centromere-linked marker, indicative of meiosis 1 segregation errors. Please see Figure S8 for raw data.

We also found that *X* chromosome NDJ increases when cohesin subunits are knocked down after meiotic S phase (Figure 5). Matα driver induced expression of an SMC1 RNAi^V22^ or SMC3 RNAi^V20^ hairpin during mid-prophase increased chromosome missegregation significantly (p = 0.011 and p<0.0001, respectively). Of particular note, SMC3 KD oocytes were extremely subfertile, and progeny were obtained in only one of three NDJ tests performed. SA KD oocytes were sterile in all NDJ tests attempted. The sterility observed when cohesin subunits are knocked down during prophase most likely stems from embryonic lethality following effective maternal depletion of these essential proteins using the germ-line optimized Valium 20 and Valium 22 expression cassettes. Regardless, the significant increase in NDJ observed when SMC1 or SMC3 is reduced indicates that new synthesis of cohesin subunits during meiotic prophase is required for accurate chromosome segregation.

To verify that meiotic cohesion is lost when SMC1 is knocked down after meiotic S phase, we used our recombinational history assay to determine the genotype of missegregating chromosomes in SMC1 KD oocytes. The number and distribution of crossovers along the *X* chromosome are normal when SMC1 KD is induced with the matα driver (Figure S8); however, although chiasmata form, they are not maintained. In two independent experiments, the majority of Diplo-*X* females arising from segregation errors harbored at least one recombinant chromosome, and segregation errors occurred primarily during meiosis I (Figures 5 and S8). These data indicate that maintenance of meiotic cohesion and chiasmata require incorporation of newly synthesized cohesin subunits into functional cohesive linkages during prophase I.

### The cohesin loader, Nipped-B, is required for cohesion rejuvenation during meiotic prophase I

We have shown that the synthesis of new cohesin subunits during prophase I is required for maintenance of cohesion and SC integrity. These data suggest that rejuvenation requires either replacement of individual cohesin subunits within pre-existing rings or the loading of new intact cohesin complexes. To further investigate the mechanism of rejuvenation, we asked whether Nipped-B, the Drosophila Scc2 ortholog [46], is required after meiotic S phase to maintain cohesion. In *S. cerevisiae*, cohesin rings form normally in *scc2* mutants but do not associate with chromosomes [47]. In Drosophila oocytes, Nipped-B co-localizes with SMC1 and SMC3 along the arms of pachytene chromosomes [20], supporting the model that loading of cohesin complexes continues to occur after meiotic S phase.

**Figure 6:**
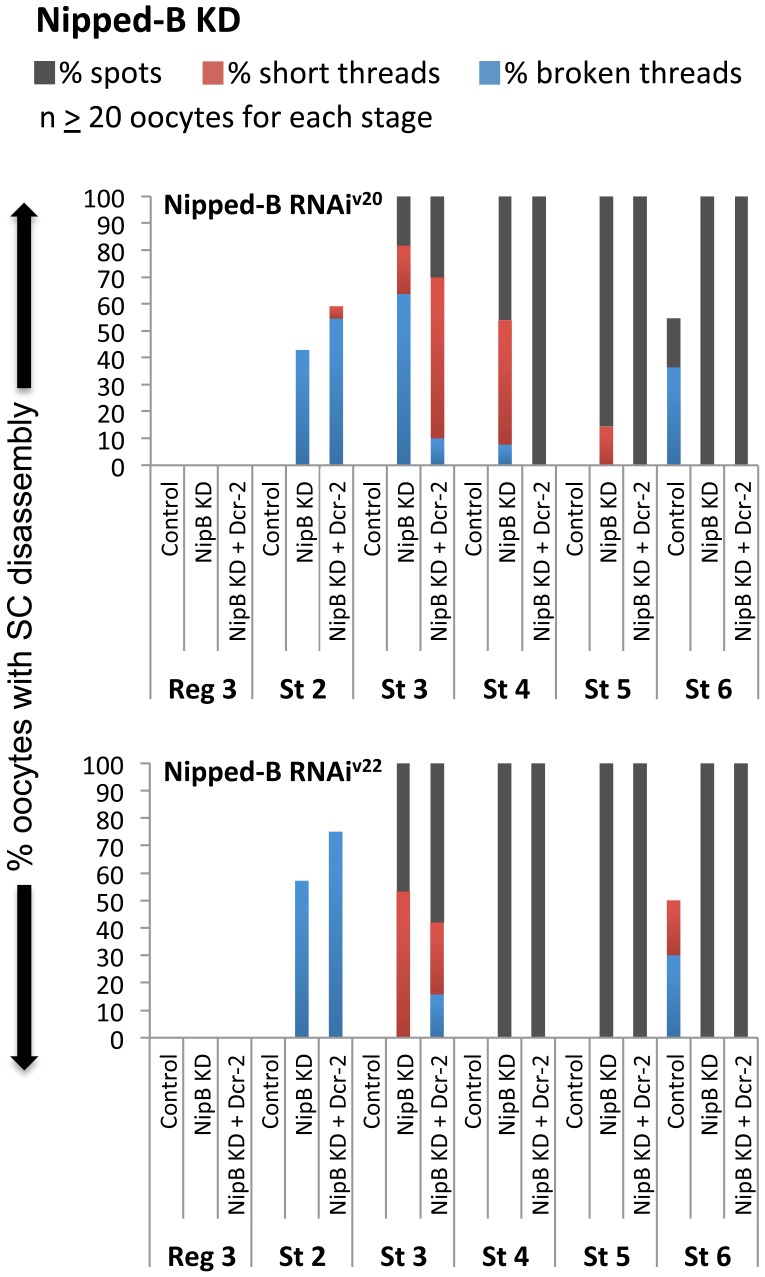
The cohesin loader Nipped-B is required after meiotic S phase to maintain the SC. Quantification of SC defects is shown for region 3 (Reg 3) through stage 6 (St 6) and two different hairpins: Nipped-B RNAi^V20^ (SH00450.N) and Nipped-B RNAi^V22^ (SH002735.N). Results are shown for oocytes from females containing the UAS-Nipped-B RNAi transgene in the absence of driver (Control), the presence of the matα driver (NipB KD), and the presence of the matα driver plus a UAS-Dicer-2 transgene (NipB KD + Dcr-2). At least 20 oocytes were scored for each genotype at each stage.

We reasoned that if loading of new cohesin rings during prophase I is required for cohesion maintenance, knockdown of Nipped-B using the matα driver would cause meiotic defects. We performed experiments using two different hairpins (Nipped-B RNAi^V20^ and Nipped-B RNAi^V22^, see Table S1) so that concerns of off-target effects could be eliminated. Figure 6 shows that when the matα driver induces expression of either of the Nipped-B hairpins, premature disassembly of the SC begins at stage 2. The observed defects are very similar for the two Nipped-B constructs, and both the percentage of oocytes affected and the severity of the defects increases during prophase I progression. If we simultaneously overexpress Dicer-2 in Nipped-B knockdown oocytes, SC defects are modestly enhanced. Importantly, the phenotypes that are manifest in Nipped-B KD oocytes closely resemble those observed when Eco or cohesin subunits are reduced during prophase I. Unfortunately, we were unable to monitor the fidelity of chromosome segregation because Nipped-B KD flies were sterile, even in the absence of Dicer-2 overexpression. However, our finding that SC defects arise when Nipped-B is reduced after S phase supports the model that cohesion rejuvenation involves the loading of new cohesin complexes, not substitution of new subunits into preexisting chromatin bound cohesin rings.

### Eco-mediated rejuvenation is independent of meiotic double-stand breaks

Under normal conditions, establishment of cohesive linkages occurs only during S phase. However, a notable exception has been described in yeast vegetative cells exposed to DNA damage during G2. In response to double-strand-breaks (DSBs), Eco1-mediated re-establishment of cohesion occurs throughout the genome during G2 [15], [17].

One possibility is that Eco-mediated rejuvenation of cohesion in Drosophila oocytes is a programmed response to the DSBs that initiate crossovers during early meiotic prophase. We set out to test this hypothesis by determining whether Eco is still required to maintain arm cohesion in the absence of meiotic DSBs. If rejuvenation of cohesion only occurs in response to DSBs, then SC defects should be absent in Eco KD oocytes that lack DSBs. In order to genetically eliminate meiotic DSBs, we utilized a null allele of the *mei-W68* gene, which encodes the evolutionarily conserved Spo11 endonuclease required for formation of meiotic DSBs [48]. In *mei-W68^1^* mutant oocytes, meiotic DSBs are eliminated and crossovers do not occur, but the temporal program of SC assembly and disassembly is normal [49].

**Figure 7:**
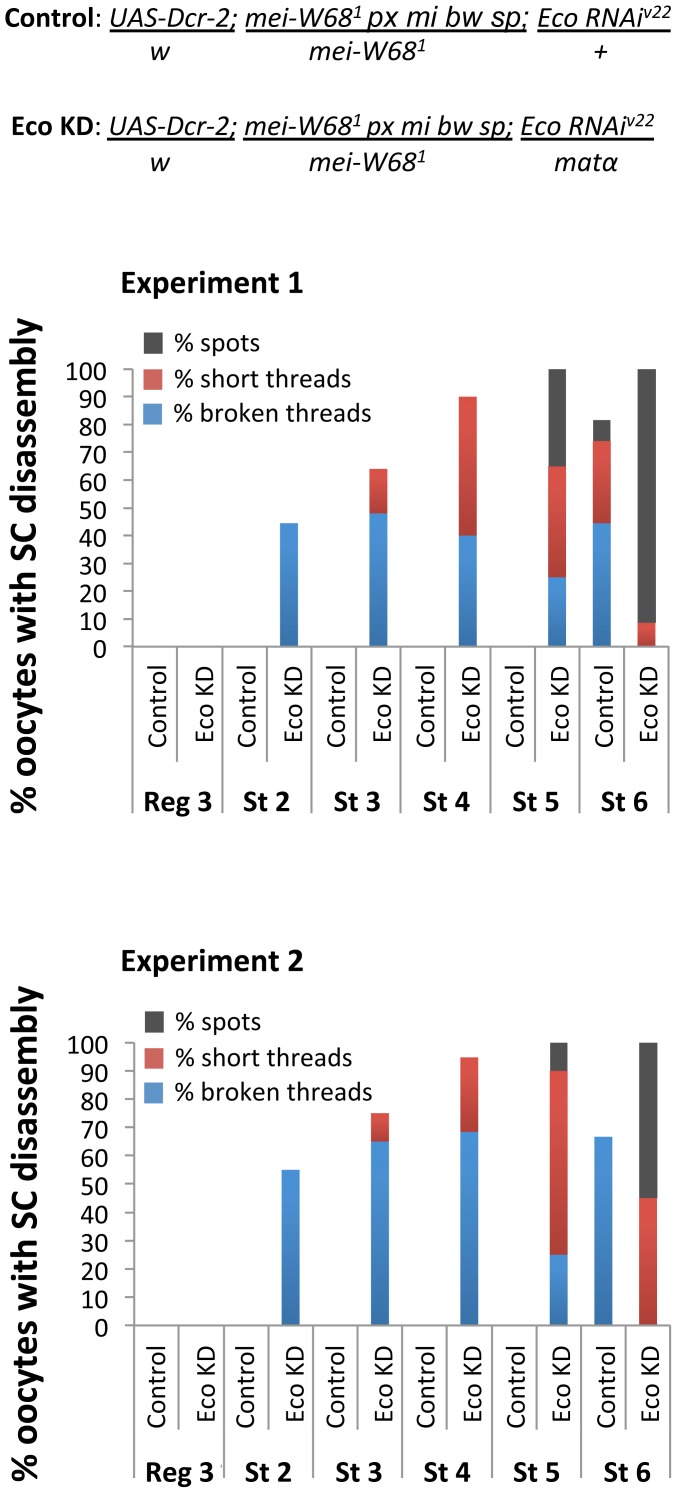
Stabilization of the SC depends on Eco even in the absence of Spo-11 induced DSBs. Quantification of SC defects in two independent experiments is presented for oocytes from region 3 (Reg 3) through stage 6 (St 6). Results are shown for oocytes from *mei-W68^1^* trans-heterozygotes (Spo-11^null^) containing the UAS-Eco RNAi^V22^ transgene and a UAS-Dicer-2 transgene in the absence (Control) or presence (Eco KD) of the matα driver. In *mei-W68^1^* Control oocytes, SC disassembly is not detected until stage 6. In contrast, premature disassembly of the SC is detectable beginning at stage 2 (St 2) in the *mei-W68^1^* Eco KD oocytes. As prophase I progresses, both the severity of the defects and the percentage of oocytes affected increase. At least 20 oocytes were scored for each genotype at each stage.

We compared the morphology of the SC in *mei-W68^1^* Eco KD oocytes (Eco RNAi^GD^ and matα driver) and *mei-W68^1^* oocytes in which the Eco RNAi^GD^ was not expressed (Eco RNAi^GD^, no driver). Two independent experiments are shown in Figure 7. We observed long, continuous SC in *mei-W68^1^* oocytes (no driver) with normal disassembly commencing at stage 6. In contrast, when Eco was knocked down in *mei-W68^1^* oocytes, premature disassembly of the SC was evident in stage 2 and became progressively more pronounced. Therefore, even in the absence of Spo-11 induced DSBs, Eco is required after meiotic S phase to maintain the integrity of the SC. These data support the model that cohesion rejuvenation during meiosis occurs though a novel mechanism that is distinct from DNA damage induced cohesion re-establishment during G2 in vegetative yeast cells.

## Discussion

Here we describe the first evidence that maintenance of meiotic cohesion during prophase I is an active process and provide mechanistic insight into this rejuvenation pathway in Drosophila oocytes. The defects that we observe when SMC1, SMC3 or SA mRNAs are knocked down after meiotic S phase indicate that newly synthesized cohesin proteins are required during prophase I for sustained cohesion until the meiotic divisions. Moreover, our finding that SC stability depends on the Scc2 ortholog Nipped-B during prophase I suggests that loading of new cohesin complexes, and not replacement of individual subunits within existing cohesin rings, occurs during prophase I. Finally, our observation that the cohesion establishment factor Eco is required after meiotic S phase argues that cohesion maintenance and chiasma stabilization require new cohesive linkages to be formed during meiotic prophase I.

Together our findings indicate that the cohesive linkages established in Drosophila oocytes during meiotic S phase are insufficient for cohesion to remain intact throughout prophase I. Accurate chromosome segregation requires more than passive endurance of the original cohesive linkages established during meiotic DNA replication. Our data support the model that cohesive linkages turn over during the protracted timeframe of meiotic prophase and that in order for oocytes to ensure levels of meiotic cohesion sufficient for accurate chromosome segregation, replacement cohesin complexes must be loaded onto the meiotic chromosomes by Nipped-B and made cohesive by the action of the acetyltransferase Eco. We use the term “rejuvenation” to describe this active process of loading cohesin complexes and generating replacement cohesive linkages during meiotic prophase I. Although it is possible that intact linkages are targeted for replacement by the rejuvenation program, we favor the model that rejuvenation acts to replace linkages that are lost due to normal turn over.

At this time, we cannot rule out the possibility that Nipped-B performs a function other than cohesin loading during meiotic prophase. However, the striking similarities between the SC defects in cohesin KD and Nipped-B KD oocytes (Figures 3 and 6) support the model that continuous loading of cohesin by Nipped-B during meiotic prophase is required for sustained cohesion and chiasma stabilization (Figure 4). Furthermore, we have previously reported that Nipped-B localizes along the arms of meiotic chromosomes during pachytene [20] and these results agree with recent reports that Nipbl, the mammalian Scc2 ortholog, localizes along the chromosome axes of meiotic chromosomes in mouse spermatocytes and oocytes [21], [22]. Interestingly, although Nipbl largely dissociates from spermatocyte chromosomes by late pachytene, it remains associated with diplotene chromosomes in mouse oocytes [21], consistent with the hypothesis that cohesin loading is required throughout the extended arrest period that mammalian oocytes undergo. Our Nipped-B and cohesin knockdown results also argue that loading of cohesin complexes onto existing lateral elements is able to occur within the context of a fully formed SC. These results nicely complement the evidence in budding yeast that the transverse filament protein Zip1 is continuously incorporated into the existing complex during pachytene [50]. Together, these results highlight the dynamic nature of the synaptonemal complex, a complex structure found almost universally in meiotic cells undergoing recombination.

How do oocytes generate new cohesive linkages outside the context of meiotic S phase? In budding yeast, cohesion can be established during G2 in response to DSBs [15], [17]. Given that induction of DSBs and initiation of meiotic recombination occur early in the meiotic program, we reasoned that cohesion rejuvenation during meiosis might be mechanistically similar to the DSB-induced re-establishment pathway in vegetative yeast cells. However, when we abolish meiotic DSBs using a null allele of *mei-W68* (Drosophila Spo-11), we find that reduction of Eco after meiotic S phase still results in premature disassembly of the SC. These results indicate that even in the absence of Spo-11 induced DSBs, an Eco-mediated rejuvenation pathway is required to stabilize the SC. Our experiments also address the possibility that meiotic DSBs initiate a signaling cascade in Drosophila oocytes that promotes conditions permissive for Eco activity outside of S phase. If this were the case, we might expect that turnover of cohesive linkages during prophase in *mei-W68* oocytes would result in premature disassembly of the SC because Eco is not active. However, we and others (Figure 7 and [49]) observe normal timing of SC disassembly in *mei-W68* oocytes that contain wild-type levels of Eco. Although we cannot rule out the possibility that a low number of Spo11-independent DSBs occur in Drosophila oocytes during prophase I, our results are consistent with the model that cohesion rejuvenation in oocytes is not a programmed response to the induction of Spo-11 triggered DSBs or their repair, and represents a novel mechanism that is distinct from that described in G2 vegetative yeast cells.

Another noteworthy example of cohesion establishment outside of S phase has been reported in budding yeast vegetative cells [18]. Under normal conditions, phosphorylation of Eco1 beginning in late S phase creates a phospho-degron recognized by the SCF ubiquitin ligase, and destruction of Eco1 prevents additional cohesive linkages from forming after S phase [19]. Although three kinases participate in this pathway [19], the initiating phosphorylation event is catalyzed by CDK1, and yeast expressing an Eco1 mutant protein that cannot be phosphorylated by CDK1 are able to establish new cohesive linkages during G2 [18]. Significantly, during prophase I in metazoan oocytes, which lasts for days (fruit flies) to decades (humans), CDK1 activity is silenced, primarily through translational inhibition of cyclins [51]. If CDK1-induced destruction of Eco1 orthologs is conserved in metazoans, inhibition of CDK1 during meiotic prophase I arrest may provide a key regulatory mechanism that allows Eco1 orthologs to remain active in metazoan oocytes beyond meiotic S phase. This would allow rejuvenation of cohesion to occur during the extended period in which oocytes must sustain a number of cohesive linkages that will be adequate to support accurate chromosome segregation.

In yeast and human cells, it is well established that the acetyltransferase activity of Eco1/Esco is required for formation of cohesive linkages [52], [53]. While Eco1-mediated acetylation of SMC3 is essential for cohesion establishment during S phase, DSB-induced cohesive linkages that are formed in G2 require acetylation of the α-kleisin cohesin subunit [54]. Although we know that Drosophila Eco is necessary for prophase I rejuvenation of meiotic cohesion, future experiments will be needed to determine whether the acetyltransferase activity of Eco is required for this process and to identify the substrate(s) of Eco1 during meiotic prophase.

Is arm cohesion more dependent on rejuvenation than centromeric cohesion in Drosophila oocytes? One interpretation of our recombinational history analyses would support this notion. However, caution is required because our assay relies solely on genetic markers and therefore only allows us to capture information about the final segregation outcome. This makes it difficult to compare our data directly with recent cytological studies of mouse oocytes that observed age-dependent weakening of centromeric cohesion prior to anaphase I [12], [14]. The clustering of Drosophila oocyte chromosomes within a compact karyosome structure during prophase I [23] precludes our ability to perform a cytological analysis similar to those for mouse oocytes. Still, from both Eco and SMC1 KD oocytes, we recovered Diplo-*X* progeny that were homozygous for the centromere-linked marker (Figures 2C, 4C, S7 and S8), indicating that at least in some cases, centromeric cohesion is definitely impacted when the rejuvenation pathway is compromised. However, most Diplo-*X* oocytes arising from knockdown of Eco or SMC1 during prophase I were heterozygous for a centromere-linked marker, consistent with disruption of arm cohesion causing loss of chiasmata and missegregation of homologous chromosomes during the first meiotic division. These results fit nicely with observations in both human and fly oocytes that bivalents with a distal crossover are more vulnerable to segregation defects [55]–[58], presumably because the closer a crossover is to the end of the chromosome, the shorter the region of arm cohesion is that holds recombinant homologs together. Interestingly, in *ord* null mutants that lack both arm and centromeric meiotic cohesion, random segregation of sister chromatids results in reductional segregation errors (homologs) that outnumber equational errors (sisters) by a factor of at least 3 to 1 [59], [60]. Therefore, our results are not inconsistent with loss of both arm and centromeric cohesion yielding a random segregation outcome. Finally, it is important to note that defects solely in centromeric cohesion prior to the first meiotic division could theoretically lead to missegregation events that yield a gamete heterozygous for a centromere-linked marker (for examples see [8], [61]). So, although our data support the conclusion that both arm and centromeric cohesion defects arise from knockdown of Eco or SMC1 in Drosophila oocytes during meiotic prophase, we cannot assign their relative contributions to the segregation errors we observe.

Our data support the model that cohesive linkages turn over in Drosophila oocytes during the normal timeframe of meiotic prophase (∼6 days) and that replacement linkages are required to ensure cohesion. However, recent studies in mouse oocytes have led to the opposite conclusion – namely, that turnover of cohesin does not occur during meiotic prophase [62], [63]. What is the basis for this apparent contradiction? One possibility is that differences in meiotic progression in fly and mouse oocytes have led to divergent mechanisms for the regulation of meiotic cohesion during prophase I. For instance, once mouse oocytes exit from dictyate arrest and mature, they complete meiosis I and remain arrested in metaphase II until fertilization. Unlike mammalian oocytes, Drosophila oocytes arrest at metaphase I and passage through the oviduct triggers resumption and completion of meiosis, even in the absence of fertilization. Perhaps the requirement to stabilize chiasmata that are under tension during metaphase I arrest requires new supplementary linkages to be formed in Drosophila oocytes but not mouse oocytes. This seems unlikely, however, given that under normal conditions Drosophila females lay fertilized eggs continuously and the metaphase I arrest usually lasts less than two hours [23].

Another possibility is that differences in the experimental tools and approaches used in the fly and mouse studies account for these contradictory results. The matα driver that we use to induce knockdown of cohesin subunits or cohesin regulators becomes active during mid-pachytene, although robust expression does not occur until late pachytene (Stage 4, Figure S1B). As such, we are manipulating Eco, Nipped-B and cohesin levels earlier during meiotic prophase than the mouse experiments that utilized the GDF-9 promoter to drive Cre recombinase and inactivate the SMC1β gene in developing oocytes [62] or those that utilized the ZP3 promoter to drive expression of TEV-resistant Rec8 during the growing phase that precedes ovulation [63]. In addition, one potential problem with ectopic Rec8 expression is that an imbalance in the normal stoichiometry of cohesin subunits may have prevented TEV-resistant Rec8 from entering the nucleus [64].

Whether rejuvenation of meiotic cohesion is a conserved feature of metazoan meiosis remains to be demonstrated. However, it is hard to comprehend why fruit flies possess a mechanism to actively keep cohesion intact during a six-day time frame if no similar program exists in mammalian oocytes during their much longer prophase I arrest. Under conditions of normal meiotic progression in Drosophila oocytes, rejuvenation ensures that the number of cohesive linkages is sufficient to promote accurate chromosome segregation. However, when Drosophila oocytes are forced to “age,” and spend approximately 20 times longer in diplotene [11], cohesion is lost prematurely and chromosomes missegregate. The observation that under “aging” conditions, the normal rejuvenation pathway is incapable of sustaining cohesion in Drosophila oocytes raises the intriguing possibility that rejuvenation becomes less efficient with age. If a meiotic cohesion rejuvenation pathway also operates in human oocytes, and its effectiveness declines with age, cohesion defects may become pronounced in older women not because the original cohesive linkages finally give out, but because the rejuvenation program can no longer supply new cohesive linkages at the same rate at which they are lost.

## Materials and Methods

### Fly stocks and crosses

Flies were reared at 25°C on standard cornmeal molasses medium. Please see Table S1 for the complete genotypes and origin of stocks used in this study. Please see Text S1 for detailed descriptions of the cross schemes utilized to generate flies for genetic and/or cytological experiments. For *X* chromosome NDJ assays, *B+* experimental females were crossed to males containing an attached *X∧Y, v f B* chromosome. In this scheme, progeny arising from normal as well as Diplo-*X* and Nullo-*X* gametes can be recovered and distinguished based on eye shape and sex. Total %NDJ and P values were calculated according to Zeng et al. [65]. *sc cv v f-y+/y* females were used to measure crossover frequency along the *X* chromosome as well as to perform NDJ tests with subsequent recombinational history analysis and male progeny were scored for each of the visible markers.

### Immunolocalization of C(3)G and SMC1 in whole-mount ovaries

Six sets of ovaries from newly eclosed females fattened overnight with extra yeast and males were dissected in 1X PBS, splayed using a tungsten needle, and fixed for 20 minutes in a mixture of 600 ul heptane and 200 ul of 2% unbuffered formaldehyde (EM grade, Ted Pella) containing 0.5% Nonidet P-40 (Surfact-Amps NP-40, Pierce). All incubations and washes were done on a rotating platform at room temperature unless otherwise noted. Ovaries were rinsed three times with 1X PBST (1X PBS with 0.2% Tween-20 (Surfact-Amps 20, Pierce)) and blocked for one hour in 1X PBS with 1% BSA. Ovaries stained only with C(3)G antibody, were incubated overnight at 4°C with primary antibody diluted in antibody buffer (1X PBS with 0.01% Tween-20 and 0.5% BSA). For SMC1 immunolocalization, ovaries were incubated in primary antibody for 2 hours at room temperature. SMC1 primary and secondary antibody incubations were completed before C(3)G primary and secondary antibody incubations were performed. Following primary antibody incubation, ovaries were rinsed three times, washed 3×20 min in 1X PBST and incubated with the appropriate secondary antibodies diluted in antibody buffer for one hour. Subsequently, ovaries were rinsed three times and washed for 20 minutes each in 1X PBST, 1X PBST containing 0.1 ug/ml DAPI, and 1X PBS containing 0.01% Tween 20. After the final wash, ovaries were separated into individual ovarioles with a tungsten needle, transferred to #1.5 18-mm poly-L-lysine-coated coverslips, and mounted in 20 µl of Prolong Gold Antifade reagent.

C(3)G mouse monoclonal antibody, clone 1A8-1G2 [38], was diluted 1∶1000 and detected using Cy3-conjugated anti-mouse secondary antibody. For simultaneous immunolocalization of C(3)G and SMC1, guinea pig polyclonal SMC1 antibody [29] was diluted 1∶2000 and detected using Cy3-conjugated anti-guinea pig secondary antibody, and C(3)G was detected using either Cy5-conjugated or Alexa Fluor-488 conjugated anti-mouse secondary. All secondary antibodies were used at a final dilution of 1∶400. Secondary antibodies conjugated to Cy3 and Cy5 were obtained from Jackson Immunoresearch Laboratories and the Alexa-488 conjugated secondary antibodies were obtained from Molecular Probes.

### Confocal analysis of the UAS-Actin-GFP reporter

To characterize the onset of expression for the matα driver, ovaries from young females containing a UASp-Actin-GFP reporter (B-071) driven by matα-Gal4-VP16 were dissected in 1X PBS, and the anterior region of each ovary splayed slightly. Ovaries were fixed for 5 minutes at room temperature in 1X PBS containing 4% formaldehyde (EM grade, Ted Pella) and rinsed three times in 1X PBS. Nuclei were stained by incubating fixed ovaries in 1 µg/ml Hoechst 33342 for 15 min, followed by a brief rinse and a 15 min wash in 1X PBS. Individual ovarioles were separated using a tungsten needle, transferred to #1.5 18-mm poly-L-lysine-coated coverslips, and mounted in 15 µl of Vectashield. Coverslips were sealed with nail polish, and slides stored at 4°C until imaging.

Images were acquired using a Nikon A1RSi laser scanning confocal controlled by NIS Elements (version 4.13). All images were collected using a 40X oil Plan Fluor DIC (NA 1.3) objective and sequential scanning mode. Single slices were captured using unidirectional scanning with a 407 nm laser (for DAPI) and 488 nm laser (for GFP).

### Single molecule FISH

The single molecule FISH probes were designed using the Stellaris Probe Designer and ordered from Biosearch Technologies (http://www.singlemoleculefish.com). The probe set consisted of a mixture of 48 DNA oligonucleotides (20 mers) complementary to the Eco open reading frame. In designing the probes, the zinc finger and acetyltransferase domains of Eco were excluded, as well as any regions with homology to non-Eco sequences within the Drosophila genome. Probes were conjugated to Quasar 570 dye.

Ovaries from 8 young females held with yeast and males for one day were dissected and slightly splayed in 1X PBS and then transferred to a 1.5 ml tube for fixation in 4% formaldehyde in 1X PBS for 15 minutes at room temperature on a nutator. After rinsing 3 times and washing twice for 5 min with 1X PBS to remove the fixative, ovaries were stored in 1 ml of 70% ethanol at 4°C for at least twelve hours. After removing the 70% ethanol, ovaries were incubated in 2X SSC containing 10% formamide for 10 min and then incubated in 100 ul of hybridization buffer (2X SSC, 10% formamide, 100 µg/ml dextran sulfate, 2 mM vanadyl ribonucleoside complex, 20 µg/ml BSA and 1 mg/ml *E. coli* tRNA) containing 50 nM probe overnight at 37°C with gentle rotation in a dark chamber. Following hybridization, all washes and incubations were performed at room temperature with rocking. Ovaries were rinsed once in 400 µl of 2X SSC containing 10% formamide, and then washed for 10 min in an additional 400 µl of the same buffer. For visual identification of germline cells, ovaries were incubated for 2 hours in a mixture of ORB mouse monoclonal antibodies, clones 4H8 and 6H4 [66] each at 1∶30 dilution in 2X SSCT (2X SCC containing 0.2% Tween 20). Ovaries were rinsed 3X and washed 3×10 min in 2X SSCT and then incubated for one hour in Alexa Fluor-488 conjugated anti-mouse secondary diluted 1∶400 in 2X SSCT. Ovaries were rinsed 3X and washed 2×10 min in 2X SSCT followed by a 20 minute incubation in 2X SSCT containing 0.1 µg/ml DAPI and an additional 10 minute wash in 2X SSCT. A tungsten needle was used to separate ovarioles before mounting on a #1 18-mm poly-L-lysine-coated coverslip with 20 µl of Prolong Gold Antifade reagent.

Imaging was performed using a Nikon A1RSi laser scanning confocal system controlled by NIS Elements (version 3.22). All images were collected using a 100X oil CFI Apochromat TIRF objective (NA 1.49) and sequential scanning mode. Single focal planes in which the oocyte nucleus was visible were captured using unidirectional scanning with a 407 nm laser (for DAPI), 488 nm laser (for ORB) and 561 nm laser (for smFISH). Control, knockdown and *eco* mutant images were acquired using the same settings and processed identically. Captured images were imported into Volocity 5.5 for quantification of mRNAs in individual germ-line cysts. ORB staining allowed each egg chamber to be cropped in order to remove the surrounding layer of follicle cells so that mRNA quantification was limited to the oocyte and nurse cells. Quantification of the number of mRNA signals was carried out using the following protocol in Volocity 5.5: First, a “find objects by % intensity” task was applied to the smFISH channel to set a threshold to best identify the bright mRNA spots. Second, a “remove noise from object, medium filter” task was applied. Then an “exclude objects by size” was added to remove the background signal from the measurements. This measurement sequence allowed determination of the total number of mRNA spots within the germ-line ROI for a single optical section. Because there is some variability in the size of egg chambers even at the same stage, the # of mRNA spots/area was used for comparison of different genotypes.

## Supporting Information

Figure S1 The germline matα driver is not active until after meiotic S phase. (A) The *P{w^+mC^ = matalpha4-GAL4-VP16}V37* transgene (matα driver) was used to induce Gal4-VP16 expression in the ovary during mid-prophase I. Onset of matα driver expression was visualized using a UAS-Actin-GFP reporter. (B) A single confocal section is shown of an ovariole from a female in which the UASp-Actin-GFP transgene is induced by the matα driver. Stages of oogenesis are noted. The matα driver is inactive in early region 2A of the germarium, the stage at which meiotic cohesion is established [67], [68]. Even with the robust expression of this reporter, the earliest matα-driven expression we observed was a relatively faint GFP signal in region 3 of the germarium, approximately 48–60 hours after oocytes undergo DNA replication [69], [70]. In the majority of ovarioles, GFP signal was first visible during stage 2 of the oogenesis (∼72 hours post-replication) and in some ovarioles, expression was not apparent until stage 3 or 4. Scale bar, 25 µm. (C) Diagram illustrates use of the matα driver to express UAS RNAi hairpin constructs in germline cells after meiotic S phase.
(TIF)

Figure S2 Eco RNAi^GD^ induced by the matα driver reduces the number of Eco germ-line transcripts but only after meiotic S phase. (A) Single molecule FISH was performed to detect Eco mRNAs (magenta) and DAPI was used to visualize DNA (blue). Because expression of the matα driver is restricted to germline cells, we used Orb staining (not shown) to distinguish germline cysts from somatic cells [66] and to define a region of interest (ROI) that included only the germline cells. In this way, we were able to quantify the number of Eco transcripts in single confocal sections of germline cysts for different genotypes. Confocal single sections are shown for germaria as well as Stage 2 and Stage 4 egg chambers from females containing the UAS-Eco RNAi^GD^ and UAS-Dicer-2 transgenes in the absence of driver (Control) and in the presence of the matα driver (Eco KD + Dcr-2). *eco^1^/eco^2^* oocytes were included to confirm that our assay is sensitive enough to detect a reduction of Eco transcripts within the germarium. The white arrow points to germarial Region 3 and the insert shows the Eco mRNA signal in Region 3. Images for each stage were captured and processed identically. Scale bars, 10 µm. (B) Quantification of Eco mRNA in Region 3, Stage 2 and Stage 4 germline cysts for Control, *eco^1^/eco^2^*, and Eco KD + Dcr-2 oocytes is shown. An unpaired t-test was performed to determine significance in relation to the control. “*” denotes significance (p<0.05). At least 10 oocytes were imaged and quantified for each genotype at each stage. In region 3, Eco transcript numbers were the same for Eco KD and control (p = 0.095), but a measurable reduction (∼14%) was observed for *eco^1^/eco^2^* compared to the control (p = 0.018). However, compared to control, Eco transcripts were decreased approximately 36% in stage 4 egg chambers (p = 0.0019) and 46% in stage 6 egg chambers (p = 0.0001) from Eco RNAi^GD^ females. Although Eco transcripts were also reduced in *eco^1^/eco^2^* females at these stages (11–12% reduction), the difference did not reach significance for either stage (p = 0.255 and p = 0.082, stage 4 and 6 respectively), perhaps because the lower concentration of Eco transcripts at later stages makes it more difficult to detect a reduction in this weak allelic combination.
(TIF)

Figure S3 Eco KD phenotypes are not due to off-target RNAi effects. (A) Schematic illustrates the three different Eco hairpins used and their targets within the Eco mRNA. Numbers within parentheses correspond to nucleotide positions of the Eco transcript. (B) Quantification of SC defects is shown for region 3 (Reg 3) through stage 6 (St 6) oocytes from females containing the UAS-Eco RNAi^V1^ and UAS-Dicer-2 transgenes in the absence (Control) and presence (Eco KD + Dcr-2) of the matα driver. At least 20 oocytes were scored for each genotype at each stage. (C) *X* chromosome NDJ increases significantly in females containing the UAS-Eco RNAi^V1^ and UAS-Dicer-2 transgenes in the presence of the matα driver (Eco KD + Dcr-2) compared to those that lack the driver (Control). 1.32 % NDJ was observed for the Control (n = 903) while 4.36% NDJ was observed for Eco KD + Dcr-2 (n = 1078). (D) Quantification of SC defects from region 3 (Reg 3) through stage 6 (St 6) is shown for oocytes from females containing the UAS-Eco RNAi^V22^ and UAS-Dicer-2 transgenes in the absence of driver (Control), the UAS-Eco RNAi^V22^ transgene and the matα driver (Eco KD) and the UAS-Eco RNAi^V22^ and UAS-Dicer-2 transgenes with the matα driver (Eco KD + Dcr-2). In control oocytes, SC disassembly is not detected until stage 6. In contrast, premature disassembly of the SC is detectable beginning at stage 3 in the Eco KD oocytes and at stage 2 for the Eco KD + Dcr-2 oocytes. As prophase I progresses, both the severity of the defects and the percentage of affected oocytes increase. When Dicer-2 is overexpressed, the phenotype is enhanced at all stages, in both the percentage of oocytes with defects and the severity of defects. At least 20 oocytes were scored for each genotype at each stage. Because females expressing matα-induced Eco RNAi^V22^ exhibited extremely low fertility (even in the absence of UAS-Dcr-2), we could not assay meiotic NDJ in this genotype.
(TIF)

Figure S4 Dicer-2 overexpression does not lead to SC defects or increased NDJ. (A) C(3)G immunostaining was performed on whole mount preparations and quantification of SC defects is shown for region 3 (Reg 3) through stage 6 (St 6) for oocytes from *y; cn bw sp* females (Control) and from females containing the UAS-Dicer-2 transgene induced by the matα driver (Matα → Dcr-2). In both Control and Matα → Dcr-2 oocytes, SC disassembly was not detected until stage 6. (B) NDJ tests were performed using *y; cn bw sp* (Control) and Matα → Dcr-2 females. No increase in NDJ was observed when Dicer-2 was overexpressed (p = 0.793).
(TIF)

Figure S5 UAS-EcoRNAi^GD^ knockdown using the Nanos-GFP-VP16 driver. (A) Projections of deconvolved Z-series are shown for C(3)G immunostaining of oocytes from females containing the UAS-Eco RNAi^GD^ transgene in the absence of driver (Control) and the presence of the nanos driver (Nanos→Eco RNAi^GD^). Normal SC appears to form in region 2A (Reg 2A) of Nanos→Eco RNAi^GD^ oocytes, but defects are apparent by region 2B (Reg 2B). Scale bar, 2 µm (B) SC defects were scored from germarial region 2A (Reg 2A) through Stage 6 (St 6) for oocytes from females containing the UAS-Eco RNAi^GD^ transgene in the absence of driver (Control) and the presence of the nanos driver (Eco KD). Normal SC disassembly commences in Control oocytes at stage 6 (St 6). In contrast, premature disassembly of the SC is detectable in Eco KD oocytes beginning at Region 2B (Reg 2B). At least 20 oocytes were scored for each genotype at each stage. (C) An *X* chromosome NDJ assay was performed on the genotypes above. NDJ was significantly higher (p<0.0001) in Eco KD oocytes (16.6% NDJ, n = 834) than in Control oocytes (1.1% NDJ, n = 1079).
(TIF)

Figure S6 Crossover frequency and distribution along the *X* chromosome are not altered when UAS-Eco RNAi^GD^ is induced with the matα driver. (A) Schematic shows the relative location of *X* chromosome visible markers used for the recombination assay. Heterochromatin is depicted by a thicker line, and a filled circle marks the centromere. (B) Meiotic crossovers were measured within four intervals in *y sc cv v f-y+/y ; P{UAS-EcoRNAi^GD^}/+ ; P{UAS-Dcr-2}/+* (Control) and *y sc cv v f-y+/y ; P{UAS-Eco RNAi^GD^}/+ ; P{UAS-Dcr-2}/P{matα-Gal4-VP16}* (Eco KD + Dcr-2) females. A two-tailed Fisher's exact test performed for each interval indicated that crossover frequency was not significantly different between Control and Eco KD + Dcr-2 oocytes. This assay was performed twice. One replicate is shown here.
(TIF)

Figure S7 The majority of Diplo-*X* progeny arising from Eco KD females result from missegregation of recombinant chromosomes during meiosis I. Two independent experiments were performed and both show similar results, depicted in graphical format in Figure 2C. The raw data obtained for each experiment is presented here. At the top of each table, the results of the initial NDJ test are provided. Diplo-*X* females were used for an additional cross to determine the recombinational history of their *X* chromosomes. Not all Dipo-*X* females resulted in sufficient numbers of progeny to enable an unambiguous genotype determination. The deduced *X* chromosome genotypes for Diplo-*X* females are listed below the NDJ results. For the first test, 9 Diplo-*X* progeny from Eco KD mothers and all of the Diplo-*X* progeny from Control mothers contained two non-recombinant (NR) *X* chromosomes; for the second test, three Diplo-*X* progeny from Eco KD mothers harbored two non-recombinant *X* chromosomes. For both tests, all other Diplo-*X* progeny inherited at least one recombinant *X* chromosome. For these, the majority (15 out of 18, and 12 out of 13) were heterozygous for the centromere-linked y^+^ marker, consistent with a meiosis I missegregation event following loss of arm cohesion and destabilization of chiasmata. In the two tests combined, four Diplo-*X* females inherited two sister chromatids (based on homozygozity of y^+^), most likely because centromere cohesion was also compromised prior to metaphase I or II.
(TIF)

Figure S8 Chiasmata are formed but not maintained when SMC1 is knocked down after meiotic S phase. (A) Crossover frequency and distribution along the *X* chromosome are normal when SMC1 is knocked down using the matα driver. Meiotic crossovers were measured within four intervals in *y sc cv v f-y+/y ; + ;P{UAS-SMC1RNAi^V22^}/+* (Control) and *y sc cv v f-y+/y ; + ;P{UAS-SMC1RNAi^V22^}/P{matα-Gal4-VP16}* (SMC1 KD) females. A two-tailed Fisher's exact test performed for each interval indicated that crossover frequency did not significantly differ between Control and SMC1 KD oocytes. (B) Diplo-*X* progeny of SMC1 KD females arise primarily from missegregation of recombinant chromosomes during meiosis I. Raw data is provided for two independent experiments that are presented in graphical format in Figure 5C. Results of each NDJ test are shown at the top with the deduced *X* chromosome genotypes for Diplo-*X* females listed underneath. In the first experiment, 5 of the 7 Diplo-*X* progeny inherited at least one recombinant *X* chromosome and in the second experiment 6 out of 7 Diplo-*X* progeny inherited at least one recombinant *X* chromosome. Of these 11 Diplo-*X* progeny, 8 contained chromosomes that were heterozygous for the centromere-linked y^+^ marker, consistent with loss of arm cohesion and chiasma destabilization causing missegregation during meiosis I.
(TIF)

Table S1 The complete genotypes of fly stocks used in this study are provided in this table as well as their origin and Bickel Lab stock numbers.
(DOCX)

Text S1 Detailed descriptions of the cross schemes utilized to generate flies for genetic and/or cytological experiments.
(DOCX)
