# Supplementary figures and images for: Rejuvenation of Meiotic Cohesion in Oocytes during Prophase I Is Required for Chiasma Maintenance and Accurate Chromosome Segregation
Source: PLoS Genet. 2014 Sep 11;10(9):e1004607. doi: 10.1371/journal.pgen.1004607 (PMC4161318; doi:10.1371/journal.pgen.1004607)

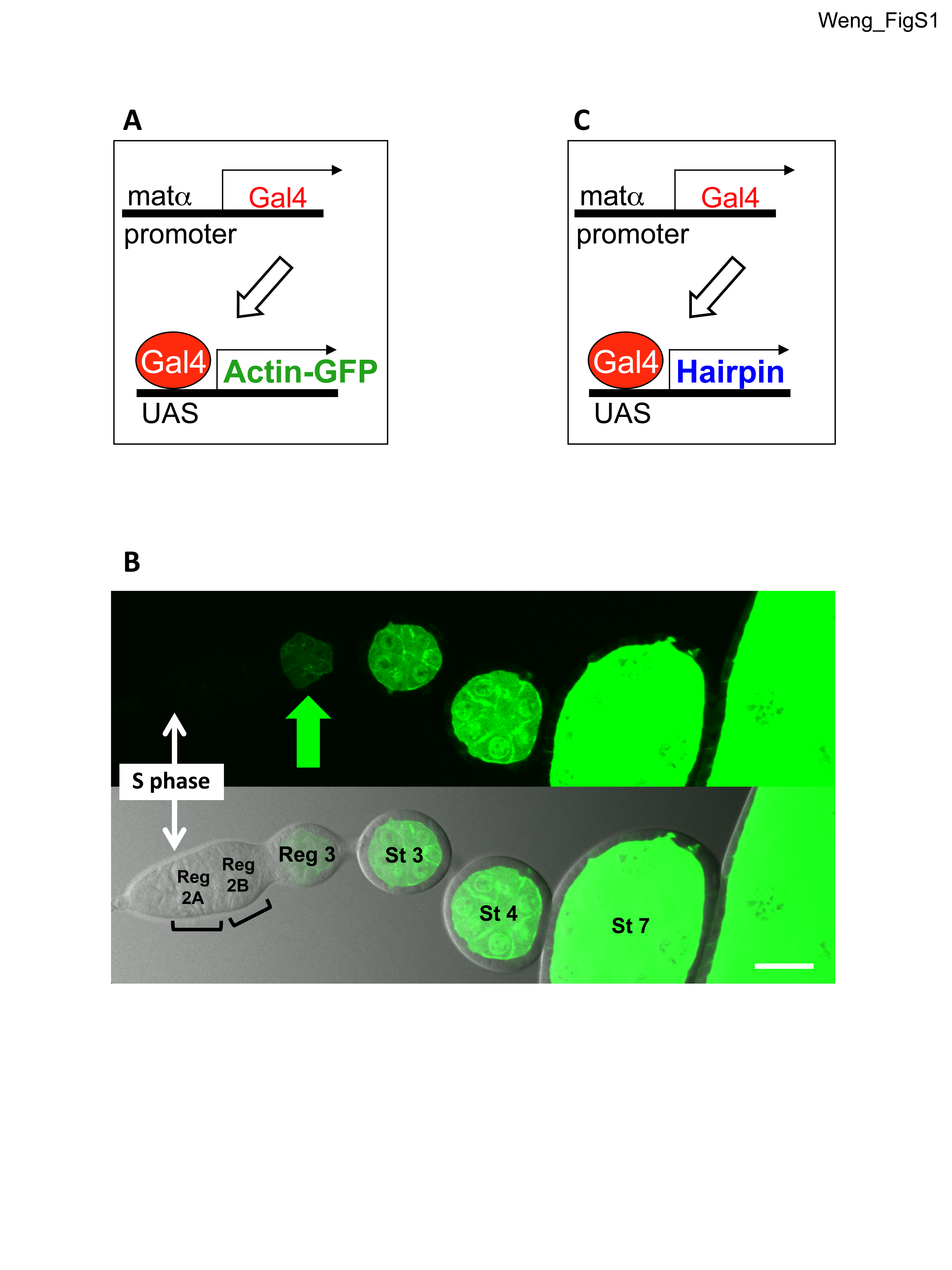

Supplement: Figure S1 — The germline matα driver is not active until after meiotic S phase. (A) The P{w+mC = matalpha4-GAL4-VP16}V37 transgene (matα driver) was used to induce Gal4-VP16 expression in the ovary during mid-prophase I. Onset of matα driver expression was visualized using a UAS-Actin-GFP reporter. (B) A single confocal section is shown of an ovariole from a female in which the UASp-Actin-GFP transgene is induced by the matα driver. Stages of oogenesis are noted. The matα driver is inactive in early region 2A of the germarium, the stage at which meiotic cohesion is established [67], [68]. Even with the robust expression of this reporter, the earliest matα-driven expression we observed was a relatively faint GFP signal in region 3 of the germarium, approximately 48–60 hours after oocytes undergo DNA replication [69], [70]. In the majority of ovarioles, GFP signal was first visible during stage 2 of the oogenesis (∼72 hours post-replication) and in some ovarioles, expression was not apparent until stage 3 or 4. Scale bar, 25 µm. (C) Diagram illustrates use of the matα driver to express UAS RNAi hairpin constructs in germline cells after meiotic S phase. (TIF) [file pgen.1004607.s001.tif]

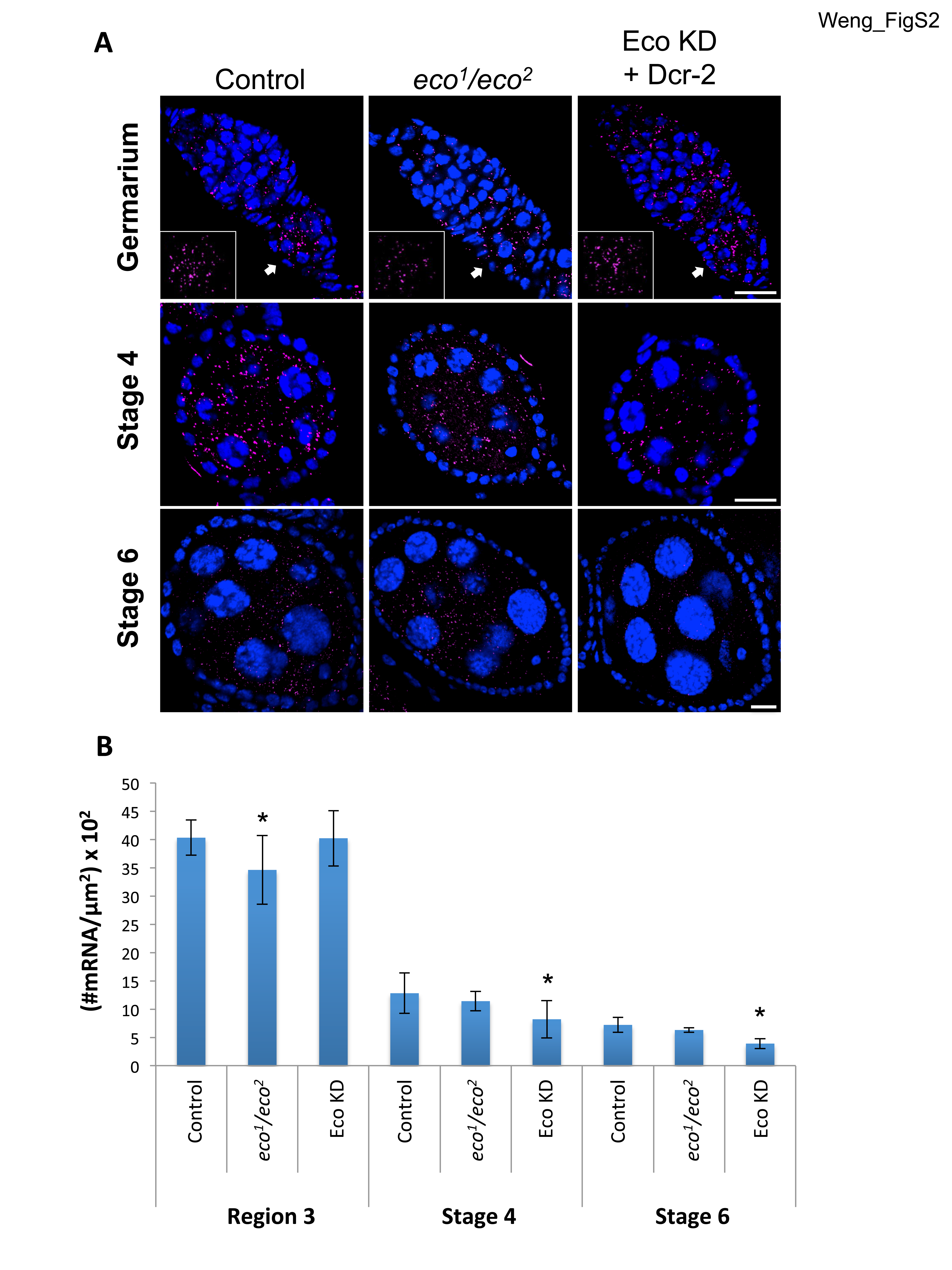

Supplement: Figure S2 — Eco RNAiGD induced by the matα driver reduces the number of Eco germ-line transcripts but only after meiotic S phase. (A) Single molecule FISH was performed to detect Eco mRNAs (magenta) and DAPI was used to visualize DNA (blue). Because expression of the matα driver is restricted to germline cells, we used Orb staining (not shown) to distinguish germline cysts from somatic cells [66] and to define a region of interest (ROI) that included only the germline cells. In this way, we were able to quantify the number of Eco transcripts in single confocal sections of germline cysts for different genotypes. Confocal single sections are shown for germaria as well as Stage 2 and Stage 4 egg chambers from females containing the UAS-Eco RNAiGD and UAS-Dicer-2 transgenes in the absence of driver (Control) and in the presence of the matα driver (Eco KD + Dcr-2). eco1/eco2 oocytes were included to confirm that our assay is sensitive enough to detect a reduction of Eco transcripts within the germarium. The white arrow points to germarial Region 3 and the insert shows the Eco mRNA signal in Region 3. Images for each stage were captured and processed identically. Scale bars, 10 µm. (B) Quantification of Eco mRNA in Region 3, Stage 2 and Stage 4 germline cysts for Control, eco1/eco2, and Eco KD + Dcr-2 oocytes is shown. An unpaired t-test was performed to determine significance in relation to the control. “*” denotes significance (p<0.05). At least 10 oocytes were imaged and quantified for each genotype at each stage. In region 3, Eco transcript numbers were the same for Eco KD and control (p = 0.095), but a measurable reduction (∼14%) was observed for eco1/eco2 compared to the control (p = 0.018). However, compared to control, Eco transcripts were decreased approximately 36% in stage 4 egg chambers (p = 0.0019) and 46% in stage 6 egg chambers (p = 0.0001) from Eco RNAiGD females. Although Eco transcripts were also reduced in eco1/eco2 females at these stages (11–12% reduction), the d [file pgen.1004607.s002.tif]

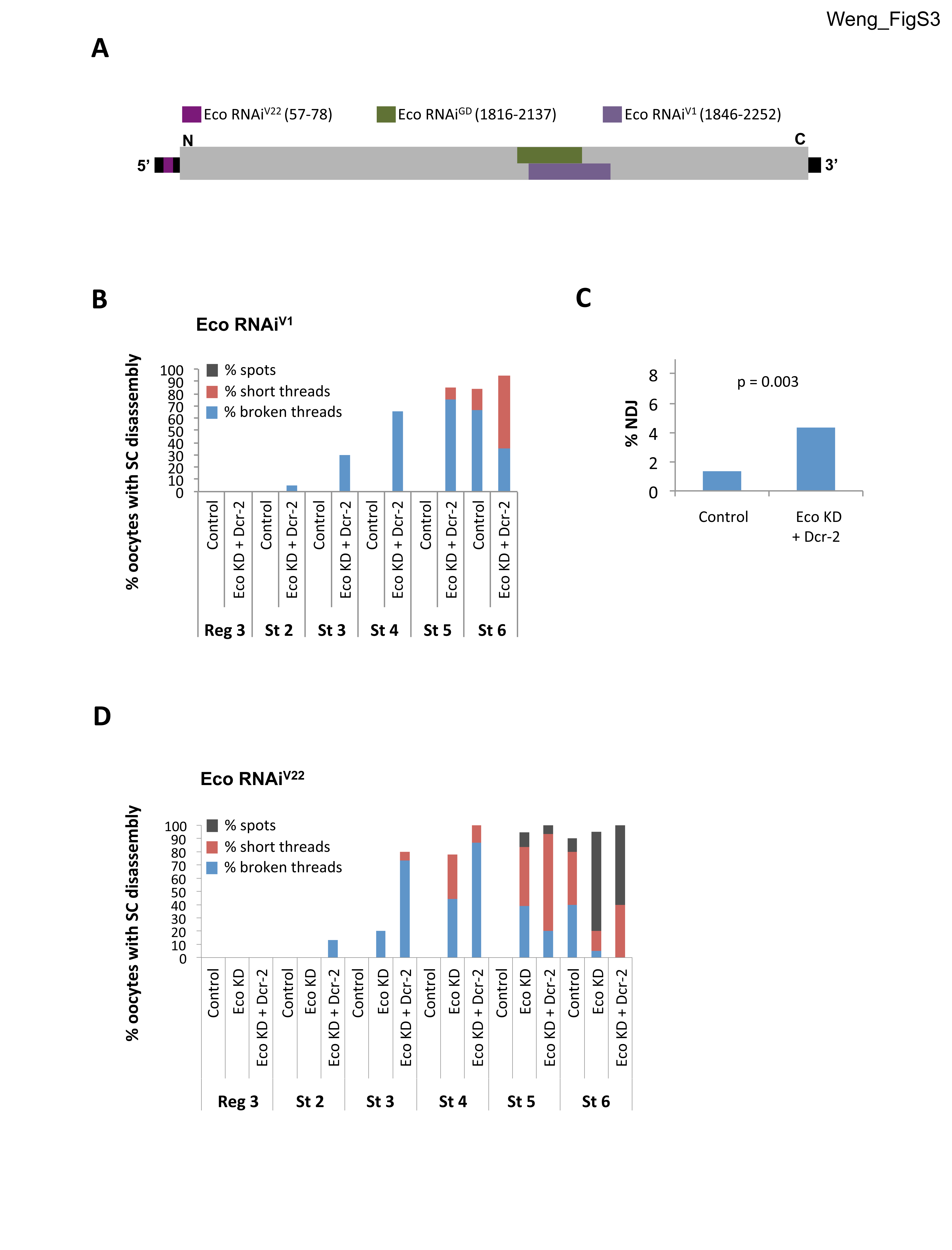

Supplement: Figure S3 — Eco KD phenotypes are not due to off-target RNAi effects. (A) Schematic illustrates the three different Eco hairpins used and their targets within the Eco mRNA. Numbers within parentheses correspond to nucleotide positions of the Eco transcript. (B) Quantification of SC defects is shown for region 3 (Reg 3) through stage 6 (St 6) oocytes from females containing the UAS-Eco RNAiV1 and UAS-Dicer-2 transgenes in the absence (Control) and presence (Eco KD + Dcr-2) of the matα driver. At least 20 oocytes were scored for each genotype at each stage. (C) X chromosome NDJ increases significantly in females containing the UAS-Eco RNAiV1 and UAS-Dicer-2 transgenes in the presence of the matα driver (Eco KD + Dcr-2) compared to those that lack the driver (Control). 1.32 % NDJ was observed for the Control (n = 903) while 4.36% NDJ was observed for Eco KD + Dcr-2 (n = 1078). (D) Quantification of SC defects from region 3 (Reg 3) through stage 6 (St 6) is shown for oocytes from females containing the UAS-Eco RNAiV22 and UAS-Dicer-2 transgenes in the absence of driver (Control), the UAS-Eco RNAiV22 transgene and the matα driver (Eco KD) and the UAS-Eco RNAiV22 and UAS-Dicer-2 transgenes with the matα driver (Eco KD + Dcr-2). In control oocytes, SC disassembly is not detected until stage 6. In contrast, premature disassembly of the SC is detectable beginning at stage 3 in the Eco KD oocytes and at stage 2 for the Eco KD + Dcr-2 oocytes. As prophase I progresses, both the severity of the defects and the percentage of affected oocytes increase. When Dicer-2 is overexpressed, the phenotype is enhanced at all stages, in both the percentage of oocytes with defects and the severity of defects. At least 20 oocytes were scored for each genotype at each stage. Because females expressing matα-induced Eco RNAiV22 exhibited extremely low fertility (even in the absence of UAS-Dcr-2), we could not assay meiotic NDJ in this genotype. (TIF) [file pgen.1004607.s003.tif]

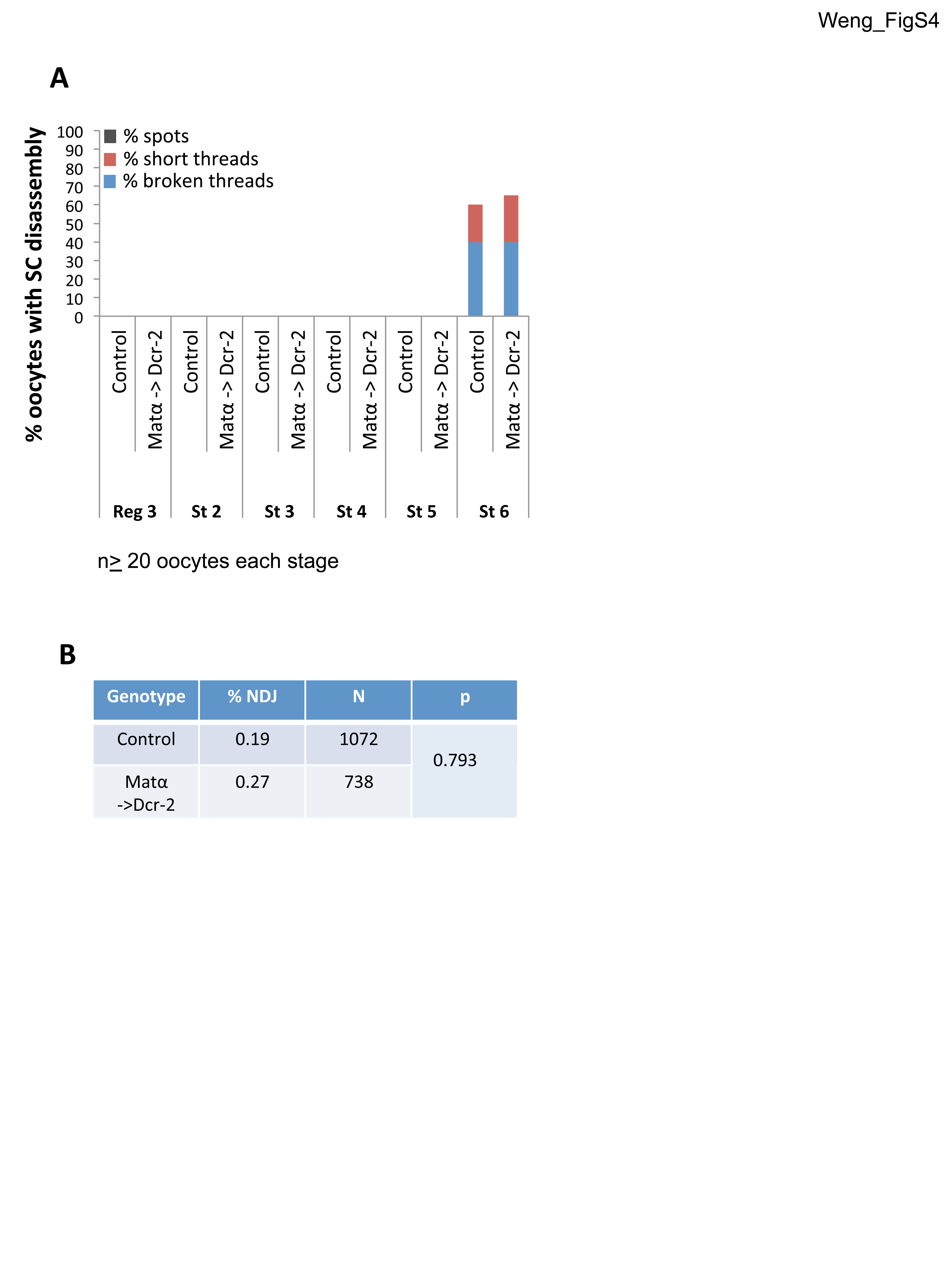

Supplement: Figure S4 — Dicer-2 overexpression does not lead to SC defects or increased NDJ. (A) C(3)G immunostaining was performed on whole mount preparations and quantification of SC defects is shown for region 3 (Reg 3) through stage 6 (St 6) for oocytes from y; cn bw sp females (Control) and from females containing the UAS-Dicer-2 transgene induced by the matα driver (Matα → Dcr-2). In both Control and Matα → Dcr-2 oocytes, SC disassembly was not detected until stage 6. (B) NDJ tests were performed using y; cn bw sp (Control) and Matα → Dcr-2 females. No increase in NDJ was observed when Dicer-2 was overexpressed (p = 0.793). (TIF) [file pgen.1004607.s004.tif]

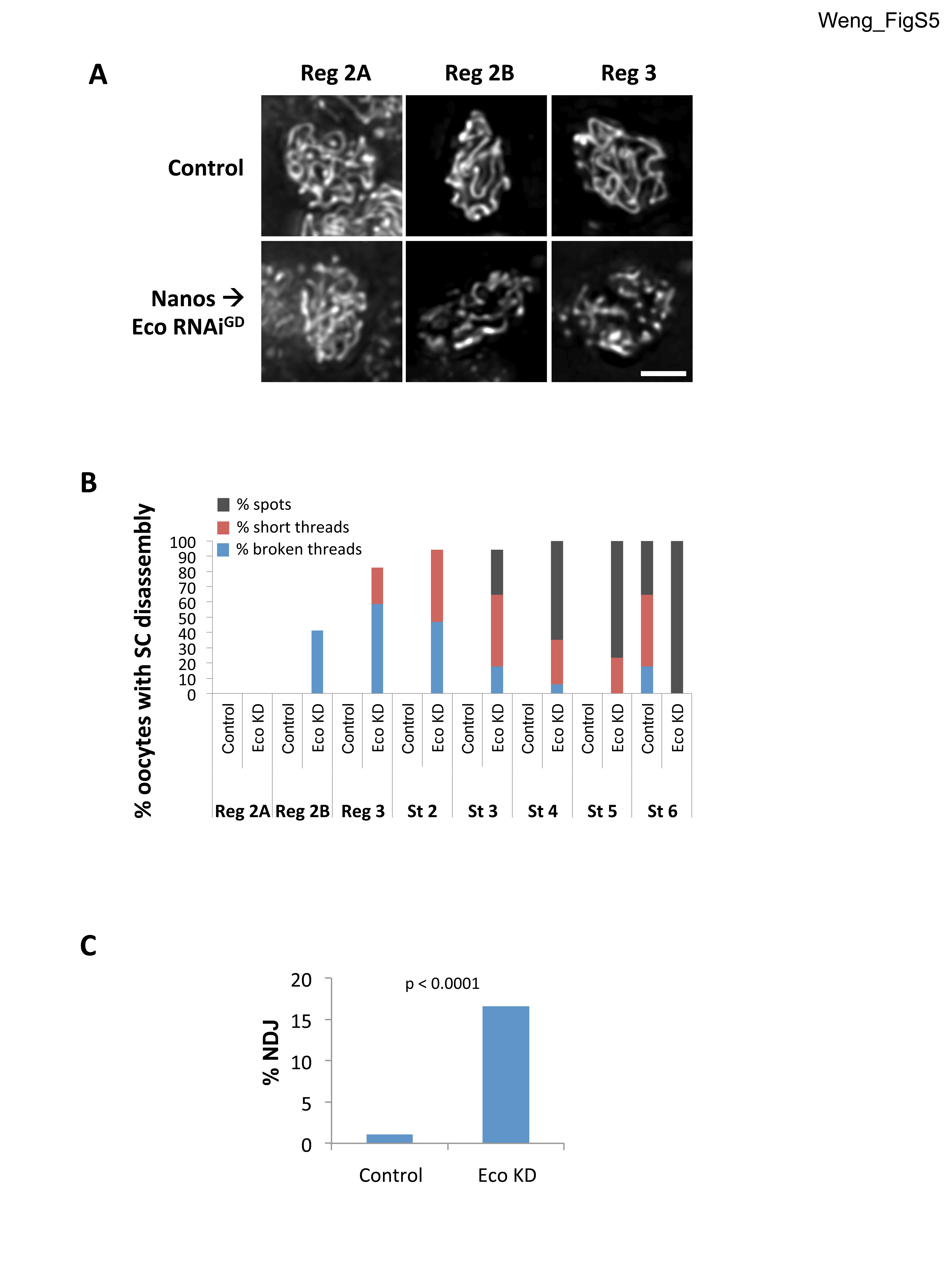

Supplement: Figure S5 — UAS-EcoRNAiGD knockdown using the Nanos-GFP-VP16 driver. (A) Projections of deconvolved Z-series are shown for C(3)G immunostaining of oocytes from females containing the UAS-Eco RNAiGD transgene in the absence of driver (Control) and the presence of the nanos driver (Nanos→Eco RNAiGD). Normal SC appears to form in region 2A (Reg 2A) of Nanos→Eco RNAiGD oocytes, but defects are apparent by region 2B (Reg 2B). Scale bar, 2 µm (B) SC defects were scored from germarial region 2A (Reg 2A) through Stage 6 (St 6) for oocytes from females containing the UAS-Eco RNAiGD transgene in the absence of driver (Control) and the presence of the nanos driver (Eco KD). Normal SC disassembly commences in Control oocytes at stage 6 (St 6). In contrast, premature disassembly of the SC is detectable in Eco KD oocytes beginning at Region 2B (Reg 2B). At least 20 oocytes were scored for each genotype at each stage. (C) An X chromosome NDJ assay was performed on the genotypes above. NDJ was significantly higher (p<0.0001) in Eco KD oocytes (16.6% NDJ, n = 834) than in Control oocytes (1.1% NDJ, n = 1079). (TIF) [file pgen.1004607.s005.tif]

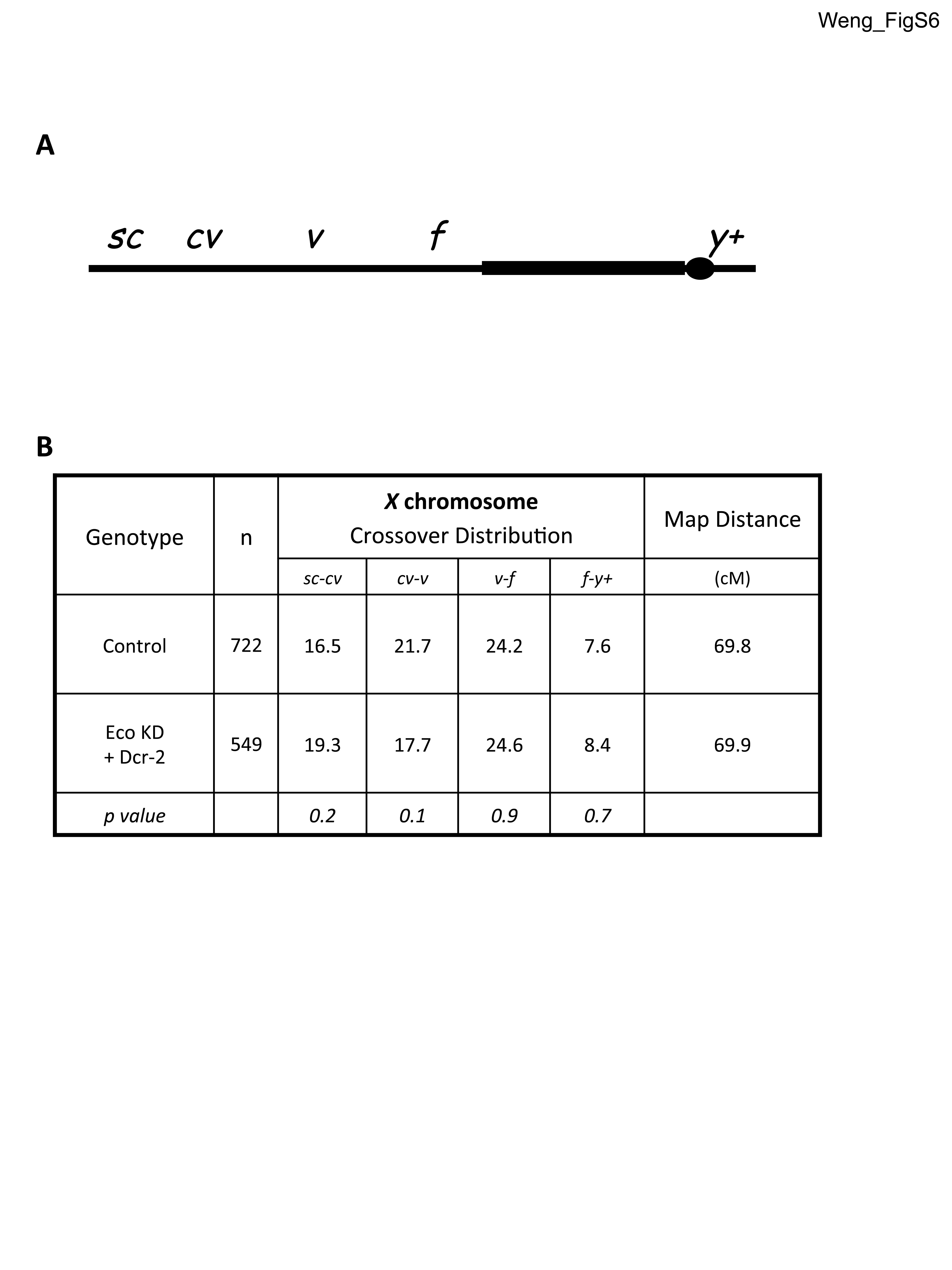

Supplement: Figure S6 — Crossover frequency and distribution along the X chromosome are not altered when UAS-Eco RNAiGD is induced with the matα driver. (A) Schematic shows the relative location of X chromosome visible markers used for the recombination assay. Heterochromatin is depicted by a thicker line, and a filled circle marks the centromere. (B) Meiotic crossovers were measured within four intervals in y sc cv v f-y+/y ; P{UAS-EcoRNAiGD}/+ ; P{UAS-Dcr-2}/+ (Control) and y sc cv v f-y+/y ; P{UAS-Eco RNAiGD}/+ ; P{UAS-Dcr-2}/P{matα-Gal4-VP16} (Eco KD + Dcr-2) females. A two-tailed Fisher's exact test performed for each interval indicated that crossover frequency was not significantly different between Control and Eco KD + Dcr-2 oocytes. This assay was performed twice. One replicate is shown here. (TIF) [file pgen.1004607.s006.tif]

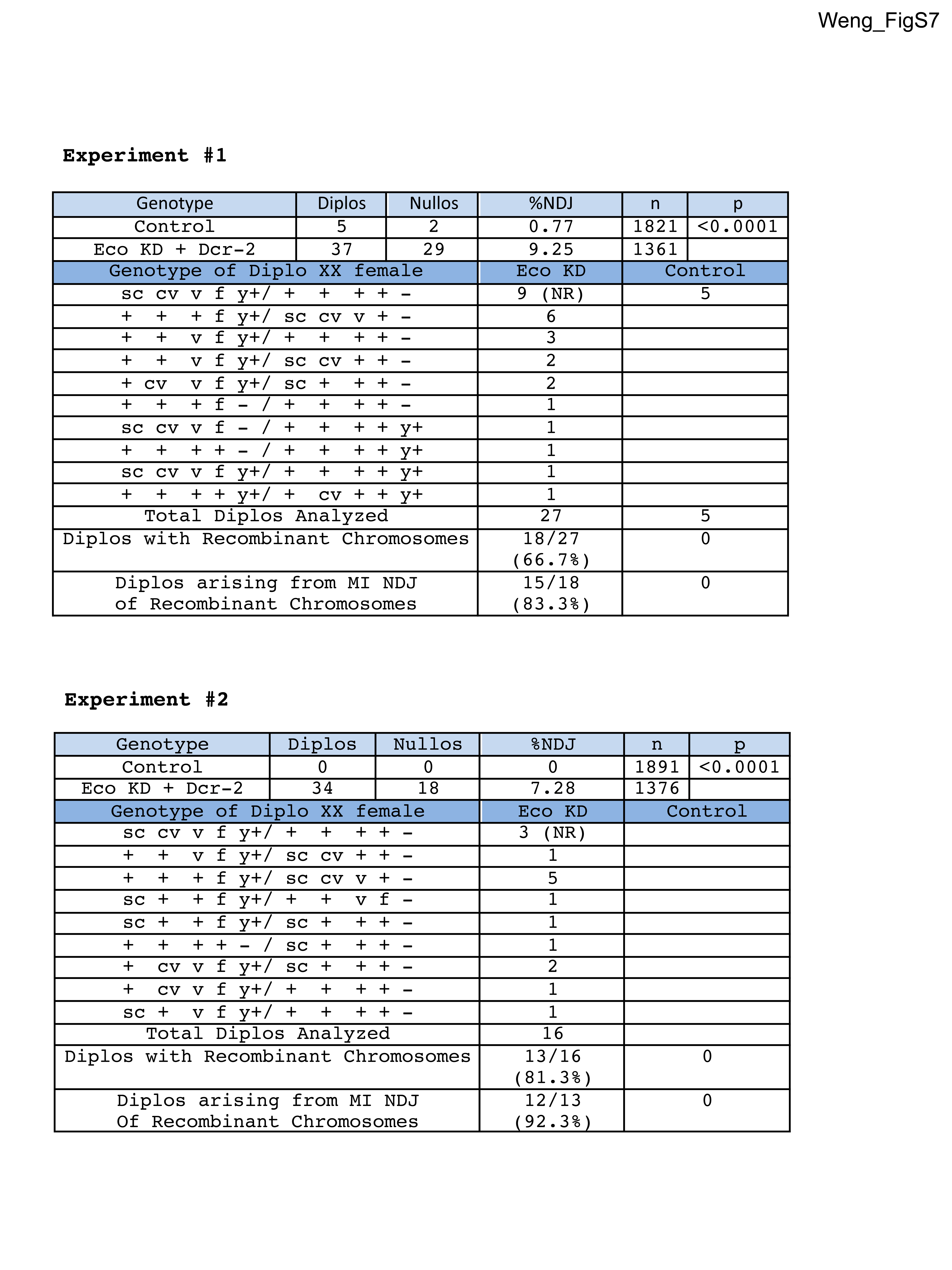

Supplement: Figure S7 — The majority of Diplo-X progeny arising from Eco KD females result from missegregation of recombinant chromosomes during meiosis I. Two independent experiments were performed and both show similar results, depicted in graphical format in Figure 2C. The raw data obtained for each experiment is presented here. At the top of each table, the results of the initial NDJ test are provided. Diplo-X females were used for an additional cross to determine the recombinational history of their X chromosomes. Not all Dipo-X females resulted in sufficient numbers of progeny to enable an unambiguous genotype determination. The deduced X chromosome genotypes for Diplo-X females are listed below the NDJ results. For the first test, 9 Diplo-X progeny from Eco KD mothers and all of the Diplo-X progeny from Control mothers contained two non-recombinant (NR) X chromosomes; for the second test, three Diplo-X progeny from Eco KD mothers harbored two non-recombinant X chromosomes. For both tests, all other Diplo-X progeny inherited at least one recombinant X chromosome. For these, the majority (15 out of 18, and 12 out of 13) were heterozygous for the centromere-linked y+ marker, consistent with a meiosis I missegregation event following loss of arm cohesion and destabilization of chiasmata. In the two tests combined, four Diplo-X females inherited two sister chromatids (based on homozygozity of y+), most likely because centromere cohesion was also compromised prior to metaphase I or II. (TIF) [file pgen.1004607.s007.tif]

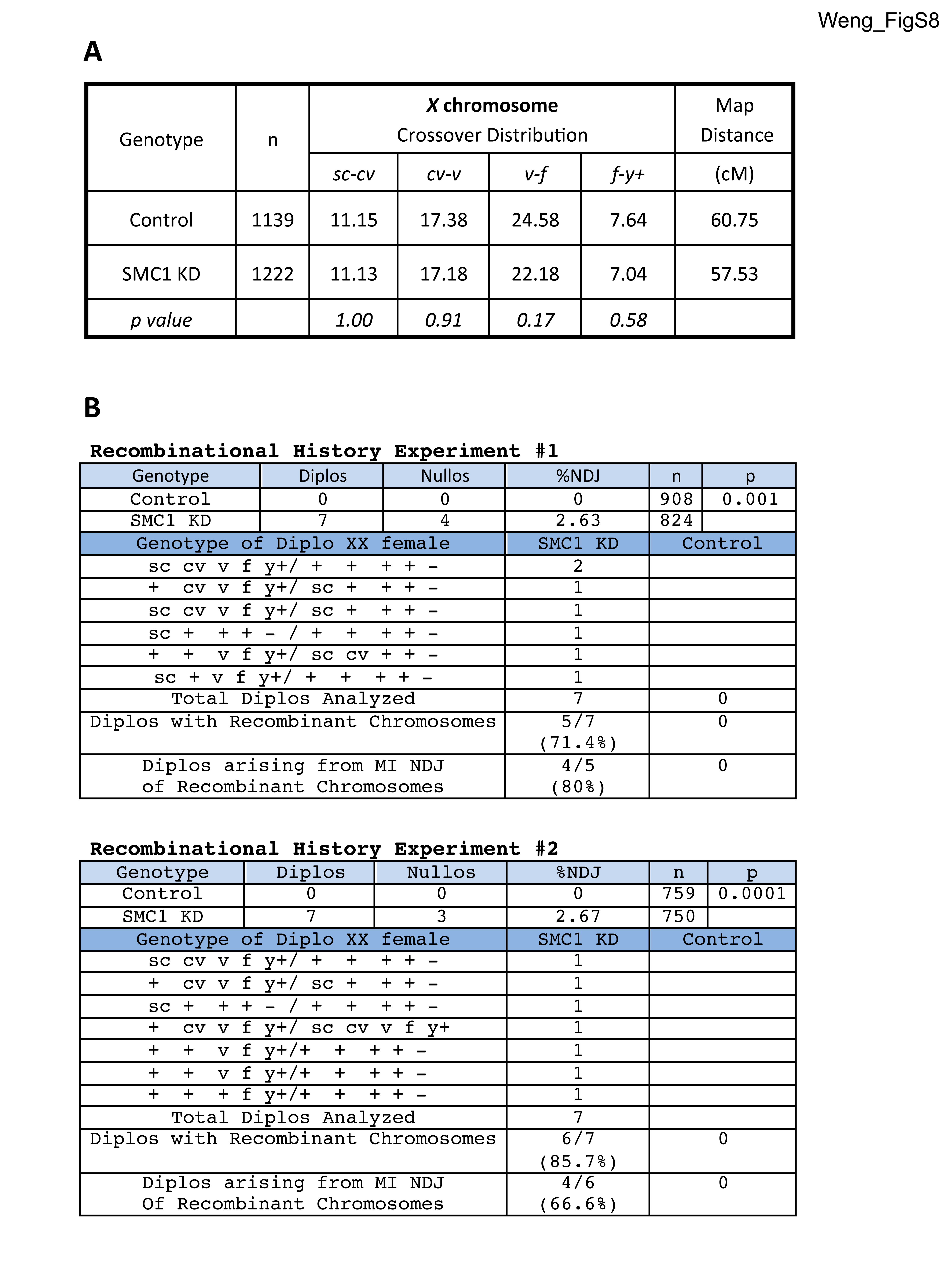

Supplement: Figure S8 — Chiasmata are formed but not maintained when SMC1 is knocked down after meiotic S phase. (A) Crossover frequency and distribution along the X chromosome are normal when SMC1 is knocked down using the matα driver. Meiotic crossovers were measured within four intervals in y sc cv v f-y+/y ; + ;P{UAS-SMC1RNAiV22}/+ (Control) and y sc cv v f-y+/y ; + ;P{UAS-SMC1RNAiV22}/P{matα-Gal4-VP16} (SMC1 KD) females. A two-tailed Fisher's exact test performed for each interval indicated that crossover frequency did not significantly differ between Control and SMC1 KD oocytes. (B) Diplo-X progeny of SMC1 KD females arise primarily from missegregation of recombinant chromosomes during meiosis I. Raw data is provided for two independent experiments that are presented in graphical format in Figure 5C. Results of each NDJ test are shown at the top with the deduced X chromosome genotypes for Diplo-X females listed underneath. In the first experiment, 5 of the 7 Diplo-X progeny inherited at least one recombinant X chromosome and in the second experiment 6 out of 7 Diplo-X progeny inherited at least one recombinant X chromosome. Of these 11 Diplo-X progeny, 8 contained chromosomes that were heterozygous for the centromere-linked y+ marker, consistent with loss of arm cohesion and chiasma destabilization causing missegregation during meiosis I. (TIF) [file pgen.1004607.s008.tif]
