# Supplementary material for: Rejuvenation of Meiotic Cohesion in Oocytes during Prophase I Is Required for Chiasma Maintenance and Accurate Chromosome Segregation
Source: PLoS Genet. 2014 Sep 11;10(9):e1004607. doi: 10.1371/journal.pgen.1004607 (PMC4161318; doi:10.1371/journal.pgen.1004607)
Supplement: Table S1 — The complete genotypes of fly stocks used in this study are provided in this table as well as their origin and Bickel Lab stock numbers. (DOCX) [file pgen.1004607.s009.docx]

**Table S1:** Fly stocks used in this study

| **Genotype** | **Hairpin ID** | **Vector** | **Abbrevi-ation** | **Source** | **Bickel Stock #** |
| --- | --- | --- | --- | --- | --- |
| **RNAi Transgenes** |  |  |  |  |  |
| *w P{VDRC.****Eco****.35982, w^+^}/C(1)DX, y w* | 13980 |  | Eco RNAi^GD^ | VDRC #35982 | V-001 |
| *y w ;*  *P{VDRC.****Eco****.35982, w^+^}* | 13980 |  | Eco RNAi^GD^ | This study | T-597 |
| *y/y^+^Y ; P{VDRC.****Eco****.35982, w^+^}* | 13980 |  | Eco RNAi^GD^ | This study | I-492 |
| *w^1118^, P{UAS-Dicer-2, w^+^}; P{VDRC.****Eco****.35982, w^+^}* | 13980 |  | Eco RNAi^GD^ | This study | T-602 |
| *y^1^ sc^1^ v^1^ ; P{y^+t7.7^ v^+t1.8^=TRiP.* ***Eco****.JF01301}attP2* | TR00759A.1 | Valium  1 | Eco RNAi^V1^ | TRiP  #31343 | H-016 |
| *y^1^ sc^1^ v^1^; P{y^+t7.7^ v^+t1.8^=TRiP.*  ***Eco****.GL00528}attP2* | SH02755.N2 | Valium 22 | Eco RNAi^V22^ | TRiP  #36789 | H-050 |
| *y^1^ sc^1^ v^1^ ; P{y^+t7.7^ v^+t1.8^=TRiP.* ***SMC3****.HMS00318}attP2* | SH00137.N | Valium 20 | SMC3 RNAi^V20^ | TRiP  #33431 | H-010 |
| *y^1^ sc^1^ v^1^ ; P{y^+t7.7^ v^+t1.8^=TRiP.*  ***SA****.HMS00272 }attP2* | SH00547.N | Valium 20 | SA RNAi^V20^ | TRiP  #33395 | H-011 |
| *y^1^ sc^1^ v^1^ ; P{y^+t7.7^ v^+t1.8^=TRiP.*  ***SMC1****.GL00558}attP2* | SH01950.N2 | Valium 22 | SMC1 RNAi^V22^ | TRiP  #36598 | H-056 |
| *y ; P{y^+t7.7^ v^+t1.8^=TRiP.*  ***SMC1****.GL00558}attP2* | SH01950.N2 | Valium 22 | SMC1 RNAi^V22^ | This study | I-503 |
| *y^1^ sc^1^ v^1^ ; P{y^+t7.7^ v^+t1.8^=TRiP.*  ***Nipped-B****.HMS00401}attP2* | SH00450.N | Valium 20 | Nipped-B RNAi^V20^ | TRiP  #32406 | H-030 |
| *y^1^ sc^1^ v^1^ ; P{y^+t7.7^ v^+t1.8^=TRiP.*  ***Nipped-B****.GL00574}attP40* | SH02735.N2 | Valium 22 | Nipped-B RNAi^V22^ | TRiP  #36614 | H-063 |
|  |  |  |  |  |  |
| **Drivers** |  |  |  |  |  |
| *w^*^ ; P{w^+mC^=matalpha4-GAL4-VP16}V37* |  |  | matα  driver | Bloomington #7063 | T-273 |
| *FM7a/y^+^Y ; + ; P{w^+mC^=matalpha4-GAL4-VP16}V37* |  |  |  | Bickel lab derivative of T-273 | T-600 |
| *w^1118^, P{UAS-Dicer-2, w^+^} ; + ; P{w^+mC^=matalpha4-GAL4-VP16}V37* |  |  |  | Bickel lab derivative of T-273 &  V-060 | T-604 |
| **Genotype** | **Hairpin ID** | **Vector** | **Abbrevi-ation** | **Source** | **Bickel Stock #** |
| *y w, P{w^+mC^=GAL4::VP16-nos.UTR}MVD2 ;*  *D/TM3, Ser Sb* |  |  | nanos  driver | Hawley lab | OL-043 |
|  |  |  |  |  |  |
| **Mutant stocks** |  |  |  |  |  |
| *y/y^+^Y ; mei-W68^1^/CyO* |  |  |  | Hawley lab | M-608 |
| *y/B^S^Y ; mei-W68^1^ px mi bw sp / SM6* |  |  |  | McKim lab | M-719 |
| *w^*^ ; mei-W68^1^ px mi bw sp/CyO; P{w^+mC^=matalpha4-GAL4-VP16}V37* |  |  |  | This study | M-831 |
| *w^1118^, P{UAS-Dicer-2, w^+^}; mei-W68^1^/CyO; P{y^+t7.7^ v^+t1.8^=TRiP.* ***Eco****.GL00528}attP2* |  |  |  | This study | M-832 |
| *eco^1^ h th st cu sr e ca/ TM3, Sb, e* |  |  | *eco^1^* | Goldberg lab | M-746 |
| *y^1^w^67c23^;P{w^+mC^y^+mDint2^=EPgy2}lark^EY00297^ eco ^EY00297^ / TM3, Sb^1^* |  |  | *eco^2^* | Bloomington  #15287 | T-329 |
|  |  |  |  |  |  |
| **Other** |  |  |  |  |  |
| *w^1118^ ; P{w^+mC^=UASp-Act5C.T:GFP}2; +/TM6C, Sb Tb* |  |  | UASp-Actin-GFP | Bloomington  #7310 | B-071 |
| *w^1118^ ; + ; P{UAS-Dicer2, w^+^}* |  |  | Dcr-2 (X) | VDRC #60009 | V-060 |
| *w^1118^, P{UAS-Dicer-2, w^+^} ; + ; Ly/ TM3,* |  |  | Dcr-2 (3) | VDRC #60010 | V-062 |
| *C(1)RM, y^2^, su(w^a^) w^a^/ X^Y,v f B* |  |  | *X^Y, vfB* |  | C-200 |
| *y sc cv v f y^+^/FM7, w ; + ;TM3,Ser y^+^ /D* |  |  |  | Hawley Lab | A-169 |
| *y sc cv v f y^+^/ FM7a* |  |  |  | Bickel lab derivative of A-169 | A-186 |
| *w*; Kr^If-1^/CyO; D^1^/TM3, Ser^1^* |  |  |  | Bloomington  # 7198 | D-309 |
| *y/y^+^Y ; cn bw sp* |  |  |  |  | I-454 |
| *y^1^ Df(1)w^67c23^* |  |  | *y w* |  | A-062 |
