# Supplementary material for: Rejuvenation of Meiotic Cohesion in Oocytes during Prophase I Is Required for Chiasma Maintenance and Accurate Chromosome Segregation
Source: PLoS Genet. 2014 Sep 11;10(9):e1004607. doi: 10.1371/journal.pgen.1004607 (PMC4161318; doi:10.1371/journal.pgen.1004607)
Supplement: Text S1 — Detailed descriptions of the cross schemes utilized to generate flies for genetic and/or cytological experiments. (DOCX) [file pgen.1004607.s010.docx]

**Detailed cross schemes used to generate flies for genetic and cytological analysis**

**Fly Stocks**

Table S1 contains complete genotypes of stocks used in this study as well as their origin and Bickel lab stock numbers (which, for clarity, are included in the detailed cross schemes provided below). The UAS-Eco RNAi^GD^ transgene in the original stock obtained from the VDRC (V-001) was hopped from the *X* chromosome to the *2^nd^* chromosome using standard techniques, and the *2^nd^* chromosome insertion was used for all experiments that utilized the UAS-Eco RNAi^GD^ transgene.

**Nondisjunction (NDJ) Assay**

Because Drosophila with certain sex chromosome aneuploidies are viable, segregation errors can be monitored by using differentially marked sex chromosomes. To measure *X* chromosome NDJ, we used the *X* chromosome dominant marker, *Bar* (*B^-^*), that affects eye shape. *B+* experimental females were crossed to males containing an attached *X^Y, v f B* chromosome (C-200). Sperm produced by *X^Y* males will contain either *X^Y, B^-^* or no sex chromosomes. Oocyte segregation errors will result in exceptional gametes that contain two *X* chromosomes (Diplo) or no *X* chromosomes (Nullo). Progeny arising from normal gametes will be *XX^Y, B^+^/B^-^* (female) or *X0, B^+^* (male) while those arising from exceptional gametes will be either *XX0, B^+^*(female) or *0X^Y, B^-^* (male)*.* Therefore, normal and exceptional progeny can be distinguished based on their sex and eye shape. Because all normal progeny survive, but half of the exceptional progeny are inviable (*XXX*^*Y* and *00*), the number of exceptional progeny was doubled and the total number of progeny was adjusted when calculating the total % NDJ. The total % NDJ and P values were calculated as described in Zeng et al. [64].

**Fly crosses**

To knock down Eco in Drosophila ovaries, males containing the UAS-RNAi hairpin transgenes (I-492, H-016, or H-050) were crossed to virgins encoding the matα (T-273) or nanos Gal4-VP16 (OL-043) drivers. To generate no-driver control females for RNAi experiments, males containing the UAS-RNAi hairpin transgenes were crossed to *y; cn bw sp* virgins (I-454). Female progeny containing both the RNAi and driver transgenes or just the RNAi transgene (no-driver control) were collected and used for genetic or cytological experiments.

To increase the Eco RNAi efficiency, males containing the UAS-Eco RNAi^GD^ hairpin transgene and the UAS-Dcr-2 transgene (T-062) were crossed to virgins encoding the matα driver (T-273). For the Eco-RNAi^V1^ and Eco RNAi^V22^ transgenes, a single cross (H-060 or H-050 males crossed to V-060 virgins) was performed to obtain males that contained both the UAS-Eco and UAS-Dcr-2 transgenes. These males were crossed to virgins encoding the matα driver (T-273). To generate no-driver control females for RNAi experiments, males containing the UAS-RNAi hairpin transgenes and the UAS-Dcr-2 transgene were crossed to *y; cn bw sp* virgins (I-454). Female progeny containing the RNAi, Dcr-2, and driver transgenes or just the RNAi and Dcr-2 transgenes (no-driver control) were collected and used for genetic or cytological experiments.

To knock down cohesin proteins (SMC1, SMC3, SA) in Drosophila ovaries, males containing the UAS-RNAi hairpin transgenes (H-056, H-010, or H-011) were crossed to virgins encoding the matα driver (T-273). Female progeny containing both the RNAi and the matα driver transgenes were collected and used for genetic or cytological experiments. To generate no-driver control females for these RNAi experiments, males containing the UAS-RNAi hairpin transgenes were crossed to *y; cn bw sp* virgins (I-454).

To knock down Nipped-B in Drosophila ovaries, males containing the UAS-RNAi hairpin transgenes (H-030 or H-063) were crossed to virgins encoding the matα driver (T-273) or virgins containing both the UAS-Dcr-2 transgene and the matα driver (T-604). Female progeny containing either the RNAi and the matα driver transgenes or the RNAi, UAS-Dcr-2, and the matα driver transgenes were collected and used for genetic or cytological experiments. To generate no-driver control females for RNAi experiments, males containing the UAS-RNAi hairpin transgenes were crossed to *y; cn bw sp* virgins (I-454).

*X* chromosome crossover frequency and recombinational history for Diplo-*X* females

In order to obtain females containing a multiply-marked *X* chromosome as well as the UAS-Dcr2, UAS-Eco RNAi^GD^ and matα driver transgene chromosomes, a number of crosses were required. Parallel crosses were performed to obtain control females that were identical except for absence of the matα driver.

*w/Y ; Kr/CyO ; D/TM3, Ser* males (D-309) were crossed to *y ; cn bw sp* virgins (I-454) and *y/Y ; cn bw sp/CyO ; D/+* male progeny were selected and crossed to *y ; P{UAS-Eco RNAi^GD^}* virgin females (I-492). *y ; {UAS-Eco RNAi^GD^}/CyO ; D/+* virgin progeny were collected and used for a subsequent cross.

*w/Y ; Kr/CyO ; D/TM3, Ser males* (D-309) were crossed to *w ; + ; P{UAS-Dcr-2}* virgin females (V-060), and *w/Y ; Kr/+ ; P{UAS-Dcr-2}/TM3*, *Ser* male progeny were selected and crossed to *y; {UAS-Eco RNAi^GD^}/CyO ; D/+* virgins (from above). *y/Y ; P{UAS-Eco RNAi^GD^}/Kr ; P{UAS-Dcr-2}/D* male progeny were selected and used in two subsequent crosses.

To generate Eco KD females, *FM7/Y ; + ; P{matα-Gal4-VP16}* males (T-600) were crossed to *y sc cv v f-y+/FM7, w ; + ; D/TM3, Ser* virgins (A-169) and *y sc cv c f –y+/FM7 ; + ; P{matα-Gal4-VP16}/TM3, Ser* virgin female progeny were crossed to *y/Y ; P{UAS-Eco RNAi^GD^}/Kr ; P{UAS-Dcr-2}/D* males (from above). *y sc cv v f-y+/y ; P{UAS-Eco RNAi^GD^}/+ ; P{UAS-Dcr-2}/P{matα-Gal4-VP16}* virgins (Eco KD females) were collected from this cross and used for subsequent genetic analyses.

To generate control females (no driver), *y/Y ; P{UAS-Eco RNAi^GD^}/Kr ; P{UAS-Dcr-2}/D* males (from above) were crossed to *y sc cv v f-y+/FM7a* virgins (A-186) and *y sc cv v f-y+/y ; P{UAS-EcoRNAi^GD^}/+ ; P{UAS-Dcr-2}/+* virgins (control, no driver) were collected and used for subsequent genetic analyses.

*sc cv v f-y+/y* Eco KD and control virgins were used to measure crossover frequency along the *X* chromosome (Figure S6) as well as to perform NDJ tests with subsequent recombinational history analysis (Figure 2B,C and S7). To measure *X* chromosome crossover frequency, *sc cv v f-y+/y* Eco KD and control virgins were crossed to *y w/Y* males (A-062), and male progeny were scored for each of the visible markers. A two-tailed Fisher’s exact test was used to determine whether differences in recombination frequency within each interval were significant. This analysis was repeated twice. One replicate is shown in Figure S6.

To determine whether the missegregating chromosomes in Eco knockdown oocytes had undergone recombination prior to missegregation, NDJ tests were performed with subsequent recombinational history analysis. *sc cv v f-y+/y* Eco KD and control females were crossed to *X^Y, v f B* males (C-200) and progeny were scored for NDJ. To genotype the Diplo-*X* progeny recovered from the NDJ test, each Diplo-*X* female was crossed to two *y w/Y* males (A-062) and the genotype of the *X* chromosomes of the Diplo-*X* female was inferred from the *sc cv v f-y+* markers in her male progeny. For a small number of Diplo-*X* progeny, we were unable to assign a definitive genotype and therefore excluded these from our final tabulation.

To determine *X* chromosome crossover frequency and recombinational history of missegregating chromosomes in SMC1 KD oocytes, the following crosses were performed. *FM7/Y ; + ; P{matα-Gal4-VP16}* males (T-600) were crossed to *y sc cv v f-y+/FM7, w ; + ; D/TM3, Ser* virgins (A-169) and *y sc cv c f –y+/FM7 ; + ; P{matα-Gal4-VP16}/TM3, Ser* virgin female progeny were crossed to *y/Y; + ; P{UAS-SMC1 RNAi^V22^}* males (I-503). The resulting

*y sc cv c f –y+/ y ; + ; P{matα-Gal4-VP16}/ P{UAS-SMC1 RNAi^V22^}* virgins were used to measure recombination frequency or to carry out NDJ tests with subsequent recombinational history analysis. Control (no driver) females for these tests (*y sc cv c f –y+/y; +; P{UAS-SMC1 RNAi^V22^}/+* were generated by crossing *y sc cv c f –y+/ Y* males to *y/y; + ; P{UAS-SMC1 RNAi^V22^}* virgins (I-503).

Eco KD in *mei-W68^1^* mutant background

To determine whether SC defects in Eco RNAi^GD^ oocytes occur in the absence of meiotic DSBs, we generated stocks that would allow us to knock down Eco in *mei-W68* mutant oocytes. Our stock construction utilized two different *mei-W68^1^* chromosomes (M-608, M-719) to eliminate any confounding issues that might arise from homozygosing extragenic modifiers or mutations on the *mei-W68* chromosome.

To generate *mei-W68^1^* Eco KD oocytes, *w^*^; mei-W68^1^ px mi bw sp/CyO ; P{matα-GAL4-VP16}* males (M-831) were crossed to *w^1118^, P{UAS-Dcr-2} ; mei-W68^1^/CyO ; P{UAS-Eco RNAi^V22^}* virgins (M-832) and *w^1118^, P{UAS-Dcr-2}/w^*^ ; mei-W68^1^/meiW68^1^ px mi bw sp ; P{UAS-Eco RNAi^V22^}/ P{matα-GAL4-VP16}* females were collected and processed for immunofluorescence.

Control oocytes for these experiments were mutant for *mei-W68*, but lacked the matα driver, and therefore contained normal levels of Eco. To generate control oocytes, *w^1118^, P{UAS-Dcr-2}/Y ; mei-W68^1^/CyO ; P{UAS-Eco RNAi^V22^}* males (M-832) were crossed to

*y ; mei-W68^1^ px mi bw sp/SM6* virgins (M-719) and *w^1118^, P{UAS-Dcr-2}/y ; mei-W68^1^/ mei-W68^1^ px mi bw sp ; P{UAS-Eco RNAi^V22^}/+* females were processed for immunofluorescence. Additional tests also were performed to verify that *mei-W68^1^* females exhibited high levels of NDJ due to lack of crossovers, and that their oocytes lacked meiotic DSBs as determined by γ-H2Av immunostaining.
